# Cadherin-11 localizes to focal adhesions and promotes cell–substrate adhesion

**DOI:** 10.1038/ncomms10909

**Published:** 2016-03-08

**Authors:** Rahul P. Langhe, Tetyana Gudzenko, Michael Bachmann, Sarah F. Becker, Carina Gonnermann, Claudia Winter, Genevieve Abbruzzese, Dominique Alfandari, Marie-Claire Kratzer, Clemens M. Franz, Jubin Kashef

**Affiliations:** 1 Zoological Institute, Cell and Developmental Biology, Karlsruhe Institute of Technology (KIT), Kaiserstrasse 12, 76131 Karlsruhe, Germany; 2 Center for Functional Nanostructures, Karlsruhe Institute of Technology (KIT), Wolfgang-Gaede-Strasse 1a, 76131 Karlsruhe, Germany; 3 Zoological Institute, Cell and Neurobiology Biology, Karlsruhe Institute of Technology (KIT), Haid-und-Neu-Strasse 9, 76131 Karlsruhe, Germany; 4 Department of Veterinary and Animal Sciences, University of Massachusetts, Amherst, Massachusetts 01003, USA; 5 Laboratory for Applications of Synchrotron Radiation, Karlsruhe Institute of Technology (KIT), Engesser Straße 15, 76131 Karlsruhe, Germany; 6 Institute for Photon Science and Synchrotron Radiation, Karlsruhe Institute of Technology (KIT), Hermann-von-Helmholtz-Platz 1, 76344 Eggenstein-Leopoldshafen, Germany

## Abstract

Cadherin receptors have a well-established role in cell–cell adhesion, cell polarization and differentiation. However, some cadherins also promote cell and tissue movement during embryonic development and tumour progression. In particular, cadherin-11 is upregulated during tumour and inflammatory cell invasion, but the mechanisms underlying cadherin-11 stimulated cell migration are still incompletely understood. Here, we show that cadherin-11 localizes to focal adhesions and promotes adhesion to fibronectin in *Xenopus* neural crest, a highly migratory embryonic cell population. Transfected cadherin-11 also localizes to focal adhesions in different mammalian cell lines, while endogenous cadherin-11 shows focal adhesion localization in primary human fibroblasts. In focal adhesions, cadherin-11 co-localizes with β1-integrin and paxillin and physically interacts with the fibronectin-binding proteoglycan syndecan-4. Adhesion to fibronectin mediated by cadherin-11/syndecan-4 complexes requires both the extracellular domain of syndecan-4, and the transmembrane and cytoplasmic domains of cadherin-11. These results reveal an unexpected role of a classical cadherin in cell–matrix adhesion during cell migration.

## Abstract (web summary)

Cadherins are typically involved in cell-cell adhesion, however cadherin-11 promotes cell migration through an undefined mechanism. Langhe *et al.* show that cadherin-11 mediates adhesion to the cell matrix at focal adhesions through interaction with syndecan-4.

## Graphical abstract

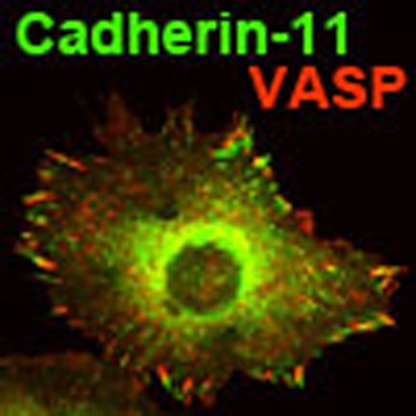

During embryonic development cell adhesion is not only important to maintain tissue morphogenesis and homeostasis, it is also crucial for processes such as cell migration, cell signalling and wound healing^1^,^2^,^3^,^4^. Importantly, dysregulation of adhesion molecules often causes developmental disorders and various diseases, including cancer and inflammation^5^. Cadherins represent a multigene family of Ca^2+^-dependent glycoproteins mediating homophilic cell–cell adhesion. Apart from forming robust cell–cell contacts, cadherins are known to initiate different intracellular signalling cascades and to modulate cell cortex tension^6^,^7^. Furthermore, different cadherins have been shown to promote cell migration^5^. In particular, the mesenchymal cadherin-11 promotes cell migration in different cell types. In humans, for instance, upregulation of cadherin-11 correlates with tumour progression and inflammatory arthritis^8^,^9^,^10^,^11^. During development cadherin-11 is also expressed in cranial neural crest cells (NCCs), a highly motile and multipotent stem-cell population giving rise to a variety of different cell types of the vertebrate face and head including cartilage, bone and ganglia^12^,^13^. In *Xenopus*, it was recently demonstrated that transient cadherin-11-mediated cell–cell adhesion is crucial for proper NCC cell migration in the context of contact inhibition of locomotion, an important mechanism for the directional migration of NCC^14^. Depletion of the homophilic adhesive function of cadherin-11 results in higher invasiveness of NCC and non-directional and incomplete NCC migration due to loss of contact inhibition of locomotion^15^. Furthermore, knockdown analysis revealed that cadherin-11 initiates filopodia and lamellipodia formation *in vivo* upstream of the guanine exchange factor Trio and small GTPases^16^. Interestingly, cadherin-11 morphant NCC lose leading edge and rear polarity, and exhibit cell rounding and membrane blebbing instead of forming cell protrusions^16^. The non-spreading and blebbing phenotype of the cadherin-11-deficient NCC raises the intriguing possibility that normally cadherin-11 plays an important role in mediating cell–substrate adhesion in migrating NCC, in addition to its classical cell–cell adhesion function.

In this study we demonstrate that cadherin-11 co-localizes with β1-integrin and paxillin to focal adhesions (FAs) in *Xenopus* NCC, where it promotes cell adhesion to fibronectin. We furthermore show that cadherin-11 also localizes to FAs in different human and murine cell lines, together with known FA markers such as paxillin, vinculin, FAK, VASP and F-actin. Moreover, cadherin-11 physically interacts with the heparan sulfate proteoglycan syndecan-4, and this interaction is required for cadherin-11-mediated adhesion to fibronectin. In rescue experiments, we furthermore demonstrate that the extracellular domain of syndecan-4, which mediates adhesion to fibronectin, and the transmembrane as well as the cytoplasmic domain of cadherin-11 are needed for proper NCC spreading and cell–matrix adhesion.

## Results

### Cadherin-11 localizes to FAs

**Figure 1:**
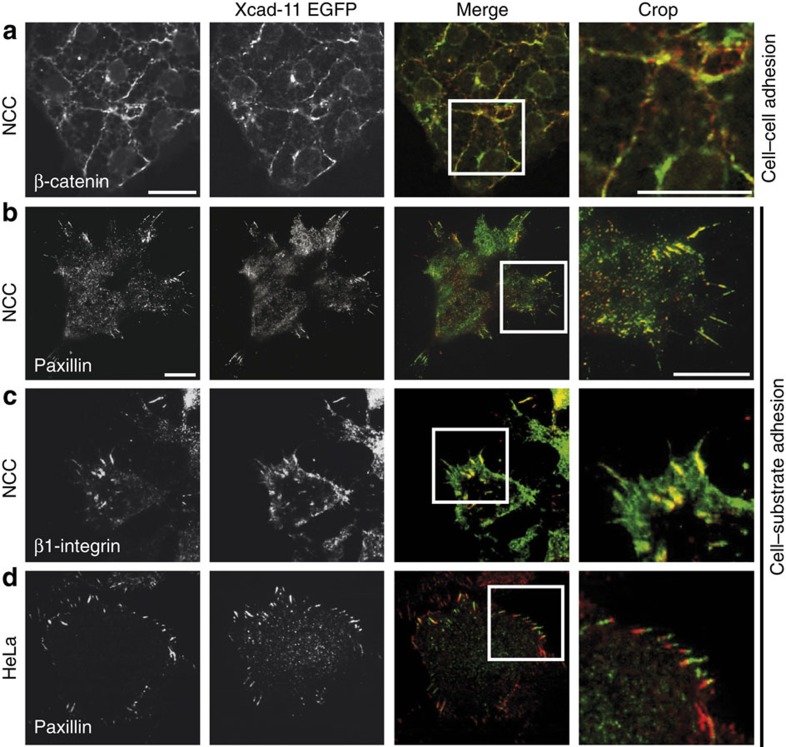
Xcad-11 is localized in focal adhesions. *Xenopus* NCC injected with Xcad-11-EGFP, explanted on fibronectin-coated glass dishes and immunostained for (**a**) β-catenin, (**b**) paxillin and (**c**) β1-integrin. (**a**) A confocal image focused on the apical side of NCC shows co-localization of Xcad-11 with β-catenin at cell–cell contacts. (**b**,**c**) TIRF images demonstrating co-localization of Xcad-11 with paxillin and β1-integrin in focal adhesions at the cell substrate. (**d**) HeLa cells transfected with Xcad-11-EGFP, immunostained for paxillin and imaged by TIRF microscopy display partial localization of Xcad-11 with paxillin at the cell substrate. Scale bars, 20 μm (**a**); 10 μm (**b**–**d**).

Cadherin-11 is a classical cadherin adhesion receptor localizing to cell–cell contacts in a variety of cell types. In *Xenopus*, expression of cadherin-11 (Xcad-11) coincides with the onset of NCC migration, pointing to an additional migration-related property of Xcad-11 (refs 12, 16). In agreement, we previously observed Xcad-11 localization to cell protrusions in migrating NCC^16^. To investigate a potential role of Xcad-11 in cell migration in more detail, we explanted Xcad-11-EGFP-injected *Xenopus* NCC on a fibronectin substrate and analysed the subcellular localization of Xcad-11 by confocal laser scanning microscopy. As expected, Xcad-11 localized to cell–cell contacts together with the adherens junction marker β-catenin (Fig. 1a). However, in addition to the apical localization at cell–cell contacts, Xcad-11 also displayed striking localization to the cell–substrate interface of NCC, as visualized by total internal reflection fluorescence (TIRF) microscopy (Fig. 1b,c). Here, Xcad-11 co-localized with paxillin (Fig. 1b) and β1-integrin (Fig. 1c) in FAs predominately at the cell periphery. These results revealed a surprising localization of a classical cadherin protein to cell–matrix contacts.

Overexpression of GFP fusion proteins can lead to aberrant subcellular localization relative to the endogenous protein. Currently there are no antibodies available for immunostaining of Xcad-11, preventing direct analysis of endogenous Xcad-11 localization in NCC. To control the potential overexpression artefacts, we re-expressed Xcad-11 at physiological levels in an Xcad-11 knockdown background. For this we co-injected an Xcad-11 antisense morpholino oligonucleotide (MO) and an Xcad-11 myc-tagged (Xcad-11-myc) rescue construct at 500 pg. In a previous titration series, we had observed full *in vivo* NCC migration at this injection dose (data not shown), indicating re-establishment of physiological Xcad-11 levels. Immunostaining against the myc-tag on explanted NCC confirmed Xcad-11 localization to FAs together with paxillin (Supplementary Fig. 1a), in agreement with our results with Xcad-11-EGFP-injected NCC.

To examine whether the atypical FA localization of Xcad-11 also occurs in other cell types, we analysed the localization of *endogenous* cadherin-11 in human foreskin fibroblast (HFF-1) cells. Again, we observed cadherin-11 co-localizing with paxillin in FAs (Supplementary Fig. 1b). Interestingly, a recent proteomics analysis also identified cadherin-11 as a FA component in HFF-1 cells^17^. In addition, transfection of Xcad-11-EGFP into HeLa (cervical cancer cells), MCF-7 (breast cancer cells) and murine NIH 3T3 fibroblasts revealed Xcad-11 localization to peripheral FA, where it co-localized with paxillin and F-actin (Fig. 1d; Supplementary Fig. 2). Likewise, HeLa cells co-transfected with Xcad-11-EGFP and vinculin-mCherry showed co-localization of both proteins at FAs (Supplementary Fig. 2a). Time-lapse fluorescence microscopy of HeLa cells showed association of Xcad-11-EGFP with newly forming FAs (Supplementary Movie 1). Kymographs generated across newly forming FA sites revealed that Xcad-11 recruitment frequently preceded vinculin recruitment (Supplementary Fig. 3), suggesting a role of Xcad-11 in the initiation of FA formation. The fluorescence still and time-lapse images also indicated partially overlapping yet distinct localization patterns of Xcad-11 and paxillin or vinculin within FAs in the mammalian cell lines. Although Xcad-11 usually localized to the distal end of FA, vinculin and paxillin typically localized to a more proximal position, with Xcad-11 and the FA markers co-localizing in the central FA region. The localization pattern of Xcad-11 at cell–matrix adhesion sites was furthermore analysed at higher resolution by structured illumination microscopy (SIM). The SIM images and intensity profile analysis confirmed partially overlapping patterns of Xcad-11 and paxillin, vinculin, FAK, VASP and zyxin within FAs (Supplementary Fig. 4). Again, Xcad-11 typically located more distally in FAs towards the cell margin, whereas paxillin, vinculin, FAK, VASP and zyxin accumulated more proximally (Supplementary Fig. 4). Xcad-11 also frequently displayed a punctate localization pattern not seen by conventional fluorescence microscopy.

Next, we asked whether other classical cadherins, such as N-cadherin or C-cadherin, also localize to FAs besides Xcad-11. We therefore examined the subcellular localization of endogenous N-cadherin together with paxillin in HFF-1 cells. As expected, N-cadherin localized to cell–cell contact sites, but not in FAs as demonstrated by TIRF microscopy (Supplementary Fig. 1c). We also transfected N-cadherin-GFP and C-cadherin-GFP into HeLa cells and immunostained against endogenous vinculin. Both N-cadherin and C-cadherin localized to cell–cell contact sites, but not to FAs (Supplementary Fig. 1d,e), indicating that FA localization is a unique property of Xcad-11 among classical cadherins. Since classical cadherins are dynamically linked via β-catenin and α-catenin to the actin cytoskeleton, we next asked whether Xcad-11 also localizes with β-catenin in FA. HeLa cell transfected with Xcad-11-EGFP and immunostained against endogenous β-catenin displayed a prominent co-localization of Xcad-11-EGFP and β-catenin at cell–cell contact sites but also in FAs (Supplementary Fig. 5). Our results are in agreement with a recent proteomics analysis identifying β-catenin and α-catenin as FA components in HFF-1 cells^17^. Interestingly, β-catenin localization to FAs requires Xcad-11 expression, since untransfected HeLa cells contain no β-catenin in cell–matrix sites. These findings support the important role of Xcad-11 in promoting cell–matrix adhesion via catenin-based linkage to the actin cytoskeleton.

FA are sites of closest cell–matrix contact. To further demonstrate Xcad-11 localization to FAs at the cell–substrate interface, adherent HeLa cells expressing Xcad-11-EGFP were inverted^18^ and basal cell surfaces were imaged by atomic force microscopy (AFM). Xcad-11-EGFP positive regions corresponded to areas of locally increased height on the basal side, indicating that Xcad-11 localized to sites of close cell–substrate contact (Supplementary Fig. 6a). The increased Xcad-11-EGFP fluorescence signal at sites of closest cell–substrate contact indicated specific cadherin recruitment, rather than locally increased plasma membrane accumulation, as a general plasma membrane marker (GAP43-mcherry) displayed homogenous localization throughout basal cell membranes in TIRF microscopy images (Supplementary Fig. 6a′′′).

To furthermore investigate whether Xcad-11 localization to FAs was specific for fibronectin substrates, HeLa cells were transfected with Xcad-11-EGFP and cultured on microstructured substrates featuring alternating fibronectin/laminin or fibronectin/BSA stripes. Xcad-11-EGFP positive FAs formed selectively on fibronectin stripes, but not on the neighbouring laminin or BSA stripes (Supplementary Fig. 6b). Specific recruitment of Xcad-11 to fibronectin-induced FA sites may explain why Xcad-11 stimulates NCC migration on fibronectin^19^.

### Xcad-11 promotes FA formation and adhesion

**Figure 2:**
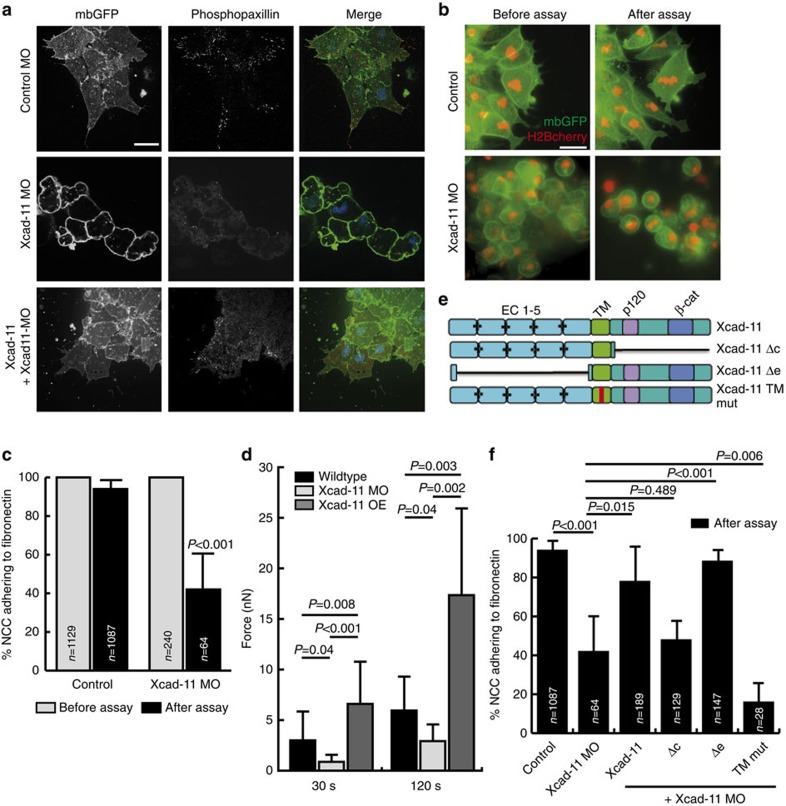
Xcad-11 promotes focal adhesion formation and cell–substrate adhesion. (**a**) NCC explants injected with mbGFP and immunostained for phosphopaxillin. Depletion of Xcad-11 by MO injection leads to loss of phosphopaxillin staining. (**b**) NCC injected with mbGFP and H2B-cherry or together with Xcad-11 MO. Fluorescence images collected at the same substrate position before (right column) and after (left column) the flipping assay. (**c**) Statistics for flipping assay (mean±s.d.), *n*=total number of cells before and after flipping assay. Results of at least three independent experiments were averaged and statistical significance was analysed by Student's *t*-test. (**d**) Adhesion forces (mean±s.d.) measured by AFM-based SCFS. At least 10 different cells were measured per condition and statistical significance was assessed according to the Mann–Whitney test. (**e**) Xcad-11 constructs for reconstitution: extracellular (EC), transmembrane (TM); (p120/ β-cat) p120-binding or β-cat-binding site. (**f**) Statistics for reconstitution (mean±s.d.), *n*=total number of cells after flipping assay. Scale bars, 20 μm.

Xcad-11 localization at the cell–substrate interface pointed towards a functional role of Xcad-11 in FA formation and/or cell–substrate adhesion. To test this hypothesis, *Xenopus* embryos were injected with control or Xcad-11 antisense MO. NCC were then explanted on fibronectin-coated dishes, fixed and immunostained for paxillin or for phosphopaxillin, a marker for active FA turnover^20^. Control MO injected NCC spread on fibronectin, formed cell protrusions and showed paxillin and phosphopaxillin staining in FAs at the leading edge (Fig. 2a; Supplementary Fig. 7). In contrast, Xcad-11 morphant NCC displayed only diffuse paxillin staining in the cytoplasm and in blebs and a complete loss of phosphopaxillin (Fig. 2a; Supplementary Fig. 7). Furthermore, Xcad-11 morphant NCC lost the ability to form cell protrusions and instead rounded-off and displayed a blebbing phenotype, consistent with our previous *in vivo* results demonstrating that Xcad-11-initiated cell protrusions are essential for proper NCC spreading and migration^16^. Co-injection of full-length Xcad-11, however, rescued paxillin and phosphopaxillin staining, further supporting a role of Xcad-11 in FA formation (Fig. 2a).

A potential function of Xcad-11 in cell–substrate adhesion was analysed using a specific flipping assay (see Methods). Wild-type NCC injected with mbGFP (to label the plasma membrane) and H2B-cherry (to label nuclei) displayed strong adhesion to fibronectin, as 94% of NCC remained on the fibronectin substrate after flipping (Fig. 2b,c). In contrast, Xcad-11 MO-injected NCC showed strongly reduced adhesion (42% after flipping, Fig. 2b,c). Since flipping assays only provide semi-quantitative results, cell adhesion was also quantitated by AFM-based single-cell force spectroscopy (SCFS, Fig. 2d). For contact times ≥10 s, adhesion forces generated by Xcad-11 morphant cells were significantly lower compared with wild-type cells, while overexpression of Xcad-11 significantly increased adhesion forces generated over wild-type NCC levels (Fig. 2d). Together, these results underline the important role of Xcad-11 in mediating FA formation and cell–substrate adhesion and suggested that Xcad-11 directly promotes adhesion to fibronectin.

We therefore sought to identify Xcad-11 domains essential for promoting cell–substrate adhesion. Classical cadherins contain three different functional domains. The extracellular part mediates cell–cell adhesion through homophilic binding^21^, while the transmembrane domain is important for lateral cadherin interactions^22^ or interactions with other transmembrane proteins^23^. The cytoplasmic tail of cadherins contains binding sites for different catenin proteins such as p120-catenin, which regulates adhesion complex assembly^24^ and lateral cadherin clustering^25^,^26^, and β-catenin, which dynamically anchors cadherins to the actin cytoskeleton via α-catenin^27^ and also interacts with vinculin^28^. To investigate the contribution of these three domains to adhesion, we designed different Xcad-11 deletion mutants as myc-tagged constructs and tested their rescue ability in the flipping assay. Before performing flipping assays, we verified proper cell-surface expression of all injected myc-tag constructs by immunostaining in *Xenopus* animal cap explants: all deletion mutants co-localized with β-catenin at the plasma membrane (Supplementary Fig. 8). In the flipping assay, Xcad-11 lacking the cytoplasmic tail (Δc) failed to rescue NCC adhesion and spreading on fibronectin (Fig. 2f; Supplementary Fig. 9), showing that the extracellular cell–cell adhesion domain of Xcad-11 alone is insufficient for cell–substrate adhesion. In contrast, Xcad-11 lacking the extracellular domain (Δe) displayed even higher rescue efficiency than full-length Xcad-11 (Fig. 2f), demonstrating that a direct interaction between the Xcad-11 extracellular domain and fibronectin is not required for promoting cell–substrate adhesion. The significant rescue ability of the Δe mutant agrees with previous observations that this mutant enhances NCC migration on fibronectin^16^,^29^. To complement the adhesion assay, we analysed the subcellular localization of the Δe-EGFP and Δc-EGFP constructs in MCF-7 cells. The Δe-EGFP mutant was often enriched at the cell edge, where it displayed the typical localization to the distal part of peripheral FAs as observed for full-length Xcad-11-EGFP, or it localized in vesicles (Supplementary Fig. 10a). In contrast, Δc-EGFP showed a fully homogeneous distribution throughout the cell without accumulation at FA sites, suggesting that the extracellular domain of Xcad-11 is not required for localization to FAs (Supplementary Fig. 10b). The transmembrane domain of different classical cadherins contains protein–protein interaction sequence-binding sites and some of these interactions can be disrupted by double-point mutations. For instance, in N-cadherin the double-point mutation significantly reduces its affinity to arcadlin^23^. In analogy, we mutated the corresponding amino acids in Xcad-11 (V506P and L507G, Xcad-11 TM mut, Fig. 2e) and analysed the rescue capacity of this construct in the flipping assay. Co-injection of Xcad-11 TM mut and Xcad-11 MO caused a more severe loss of cell–substrate adhesion compared with Xcad-11 MO alone (Fig. 2f; Supplementary Fig. 9). This suggests an important role of the Xcad-11 transmembrane domain in mediating cell–substrate adhesion. To exclude overexpression artefacts of the different Xcad-11 deletion constructs, we also injected each construct alone in wild-type *Xenopus* embryos at the same concentration used for the Xcad-11 MO rescue experiments. *In situ* hybridization for the NCC-specific marker AP2 revealed that none of the injected deletion constructs blocks NCC migration *in vivo* (Supplementary Fig. 11a,b). Furthermore, all injected NCC spread normally and formed cell protrusions when explanted onto fibronectin (Supplementary Fig. 11a). In addition, overexpression of Xcad-11-EGFP did not disturb NCC migration and cell morphology (Supplementary Fig. 11a,b).

### Xcad-11 interacts with Syndecan-4

**Figure 3:**
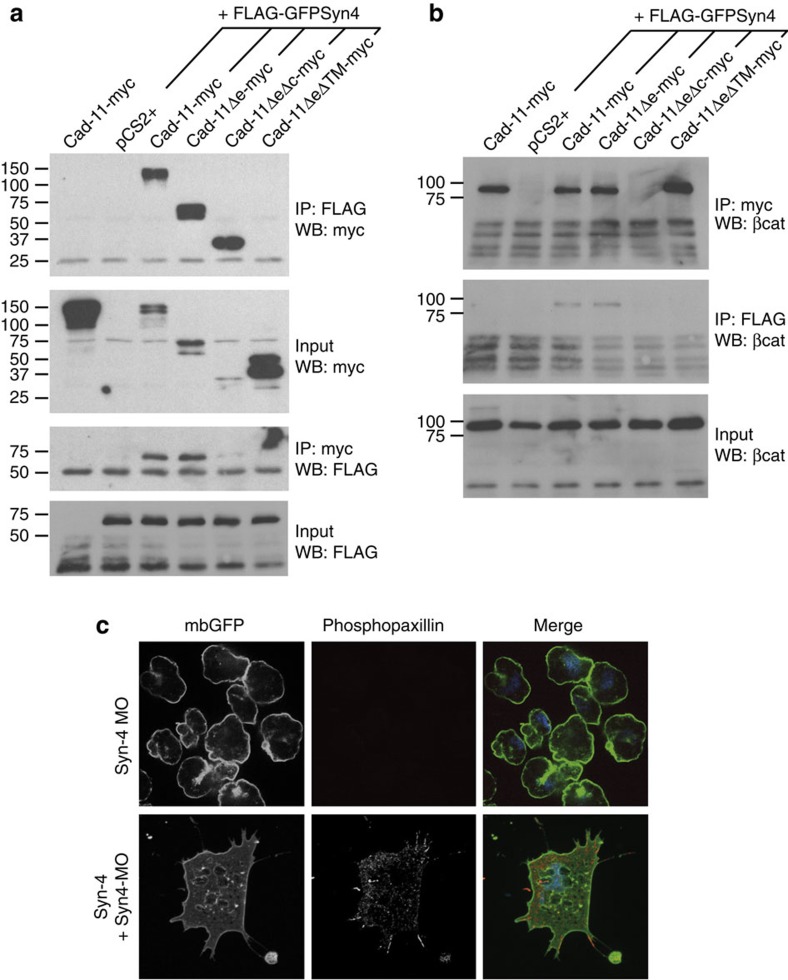
Syn-4 interacts with Xcad-11 and stimulates focal adhesion formation. (**a**) Co-immunoprecipitation of Syn-4 with different Xcad-11 constructs. (First row) Precipitation of Syn-4 with FLAG antibody from transfected HEK293 cells. Western blot for Xcad-11 (myc) showed successful co-precipitation of Xcad-11, Xcad-11 Δe and Xcad-11 ΔeΔc. In the case of Xcad-11 ΔeΔTΜ and pCS2+, no co-precipitation with Syn-4 was detected. (Second row) Input for the different Xcad-11 constructs was detected by western blotting. (Third row) Precipitation of different Xcad-11 constructs with myc antibody from transfected HEK293 cells. Western blot for Syn-4 (FLAG) showed successful co-precipitation of Xcad-11, Xcad-11 Δe and Xcad-11 ΔeΔc. In the case of Xcad-11 ΔeΔTΜ and pCS2+, no co-precipitation with Syn-4 was detected. (Fourth row) Input for Syn-4 was detected by western blotting. Transfection of Xcad-11 alone served as negative control (first panel). (**b**) Co-immunoprecipitation of Syn-4 with different Xcad-11 constructs analysed for β-catenin (βcat) binding. (First row) Precipitation of different Xcad-11 constructs with myc antibody from transfected HEK293 cells. Western blot for βcat showed successful co-precipitation of Xcad-11, Xcad-11 Δe and Xcad-11 ΔeΔTΜ . In the case of Xcad-11 ΔeΔc and pCS2+, no co-precipitation with βcat was detected. (Second row) Precipitation of Syn-4 with FLAG antibody showed successful co-precipitation of βcat when Xcad-11 or Xcad-11 Δe were co-transfected. (Third row) Input for βcat was detected by western blotting. Transfection of Xcad-11 alone served as negative control (first panel). (**c**) NCC explants co-injected with mbGFP and Syn-4 MO and immunostained for phosphopaxillin. Depletion of Syn-4 leads to loss of phosphopaxillin staining, which is rescued by co-injection of Syn-4. Scale bars, 20 μm.

There is no evidence that cadherins mediate fibronectin binding directly, but Xcad-11 may promote cell–substrate adhesion by interacting with a *bona fide* fibronectin receptor. Syndecan-4 (Syn-4) binds fibronectin and together with integrins stabilizes FA sites^30^. Syn-4 is expressed in *Xenopus* NCC and promotes directional migration of these cells^31^, and could hence act as a co-receptor for Xcad-11. To test this hypothesis, we first analysed whether Xcad-11 forms a complex and co-immunoprecipitates with Syn-4. Full-length Xcad-11 and the extracellular deletion mutant Δe were both in complex with Syn-4 (Fig. 3a). To further elucidate whether the transmembrane or the cytoplasmic domain of the Δe construct interacts with Syn-4, we designed two further deletion constructs either containing the cytoplasmic (ΔeΔTM) or the transmembrane domain alone (ΔeΔc, Supplementary Fig. 12a). Interestingly, the ΔeΔc construct interacts with Syn-4, whereas the soluble cytoplasmic domain of Xcad-11 (ΔeΔTM) does not bind (Fig. 3a). We obtained the same results when we reverse co-immunoprecipitated with Xcad-11 instead of Syn-4, indicating that Syn-4 and Xcad-11 form a stable complex (Fig. 3a). Classical cadherins interact with β-catenin, which dynamically anchors cadherins to the actin cytoskeleton via α-catenin^27^. We performed additional co-immunoprecipitation experiments to test whether β-catenin is also part of Xcad-11/Syn-4 complexes. First, we examined whether Syn-4 alone interacts with and co-immunoprecipitates β-catenin. In the absence of Xcad-11, Syn-4 did not co-immunoprecipitate with β-catenin. However, co-transfection of full-length Xcad-11 or the Δe construct together with Syn-4 recruited β-catenin to Syn-4 complexes. This interaction requires both the transmembrane and the cytoplasmic domain containing the β-catenin-binding site of Xcad-11, since co-transfection of ΔeΔTM and ΔeΔc did not cause co-immunoprecipitation of β-catenin (Fig. 3b). Taken together, these experiments identify the Xcad-11 transmembrane domain as a binding site for Syn-4 and a joint role of the transmembrane and cytoplasmic domain of Xcad-11 in promoting cell–substrate adhesion.

### Syn-4/Xcad-11 interaction promotes adhesion to fibronectin

**Figure 4:**
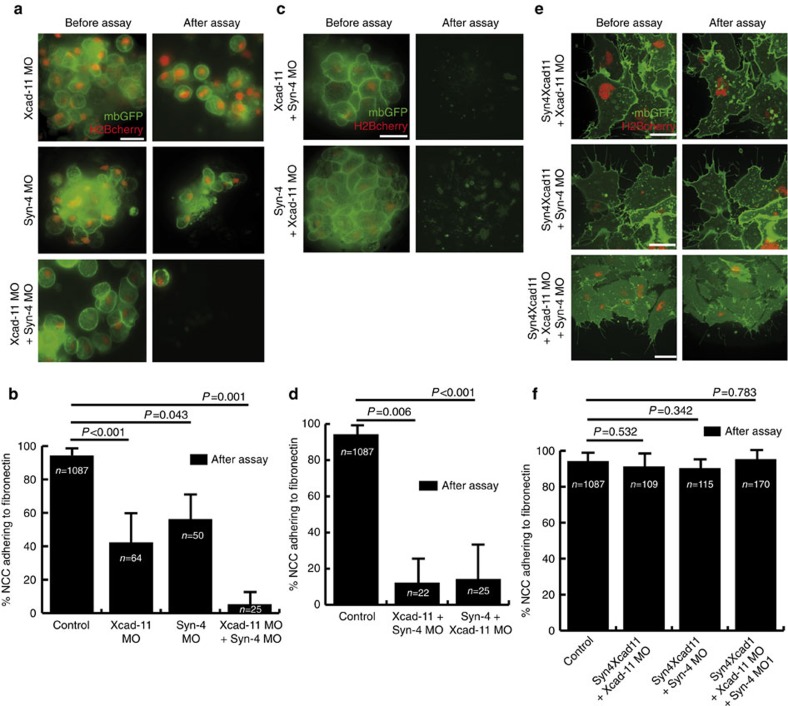
Synergistic function of Xcad-11 and Syn-4 in mediating cell-substrate adhesion. (**a**,**c**,**e**) NCC explants co-injected with mbGFP, H2Bcherry and indicated constructs before (right column) and after (left column) the flipping assay. (**b**,**d**,**f**) Statistics for flipping assays corresponding to **a**,**c** and **e**, respectively (mean±s.d.), *n*=total number of cells after flipping assay. Results of at least three independent experiments were averaged and statistical significance was analysed by Student's *t*-test. (**a**,**b**) Depletion of both Xcad-11 and Syn-4 leads to a dramatic loss of cell–substrate adhesion. (**c**,**d**) Neither co-injection of Xcad-11 nor Syn-4 rescues depletion of Syn-4 or Xcad-11, respectively. (**e**,**f**) Co-injection of chimera construct Syn4Xcad11 (Syn-4 EC domain and Xcad-11 TM as well as cytoplasmic domain) rescues cell–substrate adhesion in all NC morphant cells. Scale bars, 20 μm.

Next we determined whether Syn-4 promotes FA formation and cell–substrate adhesion in NCC. Indeed, Syn-4 morphant NCC were unable to spread on fibronectin and formed blebs instead of cell protrusions. Furthermore, Syn-4 MO-injected NCC displayed diffuse cytoplasmic paxillin staining, as well as a complete loss of phosphopaxillin staining, similar to Xcad-11 MO cells. All defects however, could be rescued by co-injection of Syn-4 in Syn-4-morphant NCC (Fig. 3c; Supplementary Fig. 7). In addition, loss of Syn-4 reduced cell–substrate adhesion (Fig. 4a,b). As knockdown of either Syn-4 or Xcad-11 resulted in a similar loss of FA formation and cell–substrate adhesion, both proteins appear to act synergistically in mediating NCC adhesion. Indeed, simultaneous loss of both proteins led to a dramatic loss of cell–substrate adhesion compared with the individual morpholino knockdowns (Fig. 4a,b). Interestingly, co-injection of Syn-4 in Xcad-11 morphants, or vice versa, did not rescue cell–substrate adhesion, indicating that both proteins act at an equal hierarchical level (Fig. 4c,d).

An equal contribution of Syn-4 and Xcad-11 in mediating NCC adhesion to fibronectin could indicate that (1) Xcad-11 and Syn-4 act independently or (2) that both proteins are in the same complex and promote adhesion to fibronectin together. To test the second hypothesis we generated a chimera construct (Syn4Xcad11) consisting of the Syn-4 extracellular domain and the transmembrane and cytoplasmic domains of Xcad-11 (Supplementary Fig. 11b). The extracellular domain of Syn-4 contains heparan sulfate chains, which are important for binding to extracellular matrix proteins such as fibronectin^32^,^33^. Strikingly, co-injection of the chimera construct rescued cell–substrate adhesion in Xcad-11, Syn-4 and double-morphant NCC cells (Fig. 4e,f). Hence, the extracellular domain of Syn-4 may promote direct adhesion to fibronectin, whereas the cytoplasmic domain of Xcad-11 could link the Syn4/Xcad-11 complex to cytoskeleton adapters at FAs. However, Syn4Xcad11-myc and Syn-4-GFP display a homogenous distribution throughout the basal cell membrane, including at FAs, but not exclusively so (Supplementary Fig. 13). Thus, the Syn4Xcad11 chimera and full-length Syn-4-GFP may share a similar localization mechanism that is not predominately targeted to FAs. It is therefore unclear whether the interaction between Xcad-11 and Syn-4 demonstrated by our co-IP experiments (Fig. 3a) only occurs at FAs or also in other membrane domains. It is tempting to speculate that the fibronectin receptor Syn-4 would recruit Xcad-11 to FAs, but additional proteins may also be required. In future it will be interesting to search for such candidates and to analyse the molecular mechanisms of Xcad-11 recruitment to FAs in more detail.

## Discussion

Our study reveals a novel function of Xcad-11 in mediating NC cell–substrate adhesion. Using high-resolution optical and atomic force microscopy, we present the first evidence that a classical cadherin localizes to FAs. Xcad-11 physically interacts with Syn-4, which mediates adhesion to fibronectin, whereas the cytoplasmic domain of Xcad-11 may bridge this complex to the cytoskeleton and drives cell migration. NCC may behave similarly to cancer cells^34^ in which cadherin-11 upregulation stimulates migration and invasiveness^8^,^9^,^10^,^35^. Since Xcad-11 localizes to FAs in different cell types, our results may uncover a general function of cadherin-11 in mediating cell–substrate adhesion and may contribute to better understanding cell migration and cell invasion in a variety of diseases.

## Methods

### Constructs

Xcad-11, Xcad-11-EGFP, Xcad-11 deletion constructs (Xcad-11 Δc and Xcad-11 Δe), Syn-4, FLAG-GFPSyn-4, GAP43-GFP, GAP43-mcherry and H2B-mcherry were described previously^16^,^29^,^31^,^36^. For Xcad-11 TM mut construct, Xcad-11 was amplified by mutagenesis PCR. The transmembrane domain of Xcad-11 was mutated by replacing two amino acids (V506P: Mut1-Fw, 5′-GTAATTTTATTAGTGATTGTGCCCTTGTTTGTGACTCTGAGGA G-3′; Rev, 5′-CTCCTCAGAGTCACAAACAAGGGCACAATCACTAATAAAATTAC-3′ and L507G: Mut2-Fw, 5′-GTGATTGTGCCCGGGTTTGTGACTCTGAGG-3′; Rev, 5′-CCTCAGAGTCACAAACCCGGGCACAATCAC-3′). For the Syn4Xcad11 construct, the Syn-4 extracellular domain was amplified by PCR with *NheI* (Fw, 5′-GCTAGCATGAGTCCGACCCTGATGT-3′) and *BamHI* (Rv, 5′-GGATCCTGTTCTCTGGAAGAATCCTTCC-3′) restriction sites. The Syn-4 extracellular domain was ligated with the transmembrane and the cytoplasmic domain of Xcad-11. For the Xcad-11 ΔeΔc construct, Xcad-11 Δe was digested with EcoRV and Acc65I to remove major parts of the cytoplasmic domain. Then, mutagenesis PCR was performed to include an in-frame EcoRI-restriction site using the following primers: Fw, 5′-GAAGAGGAAGATATCAAGCTTGGGCCC-3′′; Rev, 5′-GGGCCCAAGCTTGATATCTTCCTCTTC-3′. Xcad-11 and Syn-4 morpholino antisense oligonucleotides (Xcad-11 MO, Syn-4 MO) were designed as previously characterized^16^,^31^,^36^ and purchased from Gene Tools, LLC (Philomath, OR, USA). Vinculin-mCherry was published previously^37^.

### Embryological methods and cell culture

*Xenopus laevis* embryos were obtained by *in vitro* fertilization and staged according to Nieuwkoop and Faber^38^. RNA for injection experiments was synthesized *in vitro* using mMessage mMachine Kit (Ambion Inc.; Norwalk, CT, USA). In all, 1 ng Xcad-11-EGFP, 100 pg of Xcad-11, Xcad-11 deletion constructs with exception of Δe (50 pg), Syn4Xcad11, GAP43-GFP, H2B-mcherry, 250 pg Syn-4, 16 ng Xcad-11 MO and 16 ng Syn-4 MO were injected at the 8–16-cell stage. NC was dissected and cultured as previously described^16^. For whole-mount *in situ* hybridization, embryos were fixed in MEMFA for 1 h at room temperature and *in situ* hybridization was carried out as described before^39^. Digoxingenin-labelled antisense RNA probes were synthesized using the Digoxingenin RNA labelling Kit (Roche, Basel, Switzerland) and a template DNA encoding AP2 (ref. 40). Results of at least three independent experiments were averaged and statistical significance was analysed by Student's *t*-test. Handling of animal cap explants was performed as described previously^41^. All constructs were transcribed *in vitro* into mRNA according to the manufacture's instruction (Ambion Inc.). Except for Xcad-11-Δe (500 pg), 1 ng of the RNA constructs were injected into both blastomeres of two-cell stage embryos. HeLa and MCF-7 cells were cultured in RPMI medium, while NIH 3T3 and HFF cells were cultured in DMEM. Both media contained 10% FCS and 1% penicillin/streptomycin. HeLa, MCF-7 and NIH 3T3 cells were transfected with 2 μg of Xcad-11-EGFP using JetPEI (Polyplus Transfection SA, Illkirch, France) or TransPass (New England Biolabs, Frankfurt) and incubated on fibronectin-coated glass chamber slides for 24–48 h before fixation.

### Immunostaining

Cells were fixed with 4% paraformaldehyde in PBS for 10 min. Cells were permeabilized by washing once with nonionic surfactant (0.5% Triton-X 100 in PBS, PBST). Again, cells were washed twice with 0.1% PBST and blocked with 1% BSA/PBS solution for 30 min. After washing once with PBS, cells were incubated overnight at 4 °C with anti β-catenin (1:200; BD Biosciences, USA), paxillin (1:200; BD Biosciences, USA), phosphorylated paxillin (Y118, Cell Signalling Technology, USA), cadherin-11 (1:100, 1B4 (ref. 42)), FAK, VASP, zyxin, N-cadherin, myc (9E10, undiluted; DSHB Hybridoma Bank, USA), Xcad-11 (1:200 (ref. 16)) or β1-integrin (8C8, undiluted^43^) antibody. On the next day, cells were washed 3 × with 0.1% PBST, blocked with 1% BSA/PBS for 30 min and incubated with secondary goat anti-mouse or goat anti-rabbit Cy3 or Cy2 antibody (1:400; Dianova GmbH, Hamburg) for 1 h at room temperature, followed by three washes with 0.1% PBST. To visualize the actin cytoskeleton, cells were incubated with Alexa Fluor 568 phalloidin red (dilution 1:50 in PBS) for 30 min at room temperature. Animal cap explants were cultured on BSA-coated Petri dishes and fixed in 4% paraformaldehyde buffered in PBS modified to amphibian osmolarity (2.7 mM KCl, 0.15 mM KH_2_PO_4_, 103 mM NaCl and 0.7 mM NaHPO_4_, pH 7.5) for 30 min at room temperature. For fluorescence immunohistochemistry, animal caps were permeabilized by incubation in 20% DMSO/80% methanol overnight at −20 °C. After rehydration with descending methanol concentrations, the explants were washed extensively with APBS and incubated with blocking buffer (20% horse serum in APBS) for 2 h at room temperature. Incubation with the primary antibodies mouse anti myc (9E10, undiluted supernatant) or anti XB-cadherin (6D5, undiluted supernatant) and rat anti-β-catenin (PGDS 7D12, undiluted supernatant; provided by R. Rupp) was performed overnight at 4 °C. After washing extensively with APBS and reincubation with blocking buffer for 1 h at room temperature, the secondary antibody anti-mouse Cy3 and anti-rat Cy2 (Dianova; 1:200 in blocking buffer), respectively, was applied overnight at room temperature. The explants were counterstained with DAPI to visualize the nuclei, washed extensively in APBS, and finally embedded with Mowiol-DABCO.

### Flipping assay

NCC explants were performed as previously described^16^. Several NCC explants were analysed and counted using a spinning disc confocal (Zeiss Cell Observer, × 63 oil objective, AxioCam MRm camera) or fluorescent microscope (Leica DMIR2, × 63 oil objective, Electronicbox Leica CTR MIC, Digital camera C4742-95-12 ERG). The precise position of each explant was recorded based on the *x/y*-stage positioning scale of the spinning disc microscope. Afterwards, the flipping assay was performed. The chamber-slide containing the NCC explants was completely filled with Danilchick's buffer. The chamber slide was then carefully lifted and dipped in a large vessel also containing Danilchick's buffer. The slide was then rotated by 180° and held in an upside-down position for 15 min, rotated back 180° and carefully removed from the large vessel. After the flipping procedure, NCC explants were imaged at the same positions as before the assay. Finally, the percentage of NCC adhering to the substrate before and after the assay was calculated. At least three different explants were measured per condition. Statistical analysis was performed using the Student's *t*-test.

### AFM-based SCFS

AFM-based SCFS experiments were performed using a NanoWizard II AFM (JPK) incorporating a JPK CellHesion module with an extended vertical pulling range of 100 μm. All measurements were performed at room temperature using tipless V-shaped cantilevers with a nominal spring constant of 0.06 N m^−1^ (NP-O, Bruker). To facilitate cell capture, cantilevers were functionalized with concanavalin A^44^. A single-cell suspension was prepared from dissociated CNC explants as described above and pipetted into a plastic tissue culture Petri dish functionalized with fibronectin and containing 2 ml pond water. The spring constant of the cantilever was determined *in situ* using the thermal noise method^45^. A single cell was then immobilized on the cantilever by approaching the cell with a contact force of 0.5 nN for 3 s. After a recovery period of 5–10 min, SCFS measurements were performed using a 1.5-nN contact force, a 5 μm per second approach and retract speed and a contact time ranging from 5 to 120 s. Usually several force curve per contact time and cell were recorded. The cell–substrate contact position was changed after each force cycle. Maximum detachment forces were extracted from the recorded force curves using the JPK IP software.

### Cell inversion procedure

For scanning the basal cell side of adherent cells by AFM, HeLa cells were transfected with Xcad-11-EGFP using FuGene HD (Roche) and incubated on fibronectin-coated PDMS for 16 h. Afterwards, cells were fixed with 4% PFA and inverted according to a previously published protocol^18^. The basal cell side was scanned using a JPK Nanowizard II AFM mounted on a Zeiss Axiovert optical microscope. Imaging was performed in PBS in contact mode with gold-coated silicon nitride V-shaped cantilevers (MLCT-C) with a spring constant of 0.06 N m^−1^ (Bruker). After AFM scanning, fluorescence images of cells were collected and correlated with the corresponding AFM height images. AFM images were processed using the JPK IP software and the fluorescence images were analysed with ImageJ (http://rsbweb.nih.gov/ij/).

### Superresolution SIM

Superresolution structured illumination microscopy (SR-SIM) was performed with a nonserial Elyra PS.1 (www.zeiss.com) prototype using a × 63/1.40 oil DIC objective and appropriate emission filters (transmission of 500–550 nm for 488 nm, or double bandpass 420–545 nm and 575–640 nm for 561 nm excitation, respectively). Detection was performed with an Andor iXon 885 EMCCD camera. Twenty-five frames of raw data (five rotations and five shifts per rotation of the grid) were acquired and final images calculated with the Zeiss ZEN software. Multicolour SIM images were aligned by acquiring images of multifluorescent 200 nm beads mounted on slides and calculating colour shift with an algorithm implemented in the Zeiss software. Intensity profiles were measured with Zeiss ZEN software and exported to Microsoft Excel.

### Co-immunoprecipitations

Co-immunoprecipitation experiments were performed in HEK293 obtained from ATCC and transfected with XtremeGENE HP (Roche) according to the manufacturer's instructions. Forty-eight hours after transfections, total cellular protein was extracted in RIPA (25 mM Tris-HCl pH 7.6, 150 mM NaCl, 1% NP-40, 1% sodium deoxycholate, 0.1% SDS) with 5 mM EDTA and 1 × Halt Protease and Phosphatase Inhibitor Cocktail (Thermo Scientific) and immunoprecipitated for 2.5 h at room temperature using either the mouse anti-myc (9E10) or the mouse anti-flag (M2, Sigma) antibody. Immunoprecipitates were washed three times for 5 min in RIPA and eluted in reducing Laemmli buffer. Proteins were detected by western blot using the antibodies 9E10, M2 (1:4,000), or rabbit anti-β-catenin (Abcam, 1:4,000).

### Microstripe assay

Fibronectin/BSA and fibronectin/laminin stripe patterns were produced on glass surfaces by microcontact printing^46^. For stamp fabrication, polydimethylsilfoxane (PDMS, Sylgard, www.dowcorning.com) was mixed with cross-linker at a ratio of 10:1, degassed in a vacuum chamber and added onto a micropatterned silicon master for 4 h at 60 °C. The PDMS stamp was peeled off the master, cleaned with ddH_2_O, then with 100% ethanol and afterwards dried with nitrogen gas. BSA (50 μg μl^−1^) or laminin (20 or 50 μg μl^−1^) solution was applied onto the PDMS stamp and incubated for 30 min at room temperature (BSA) or for 1 h at 37 °C (laminin) in a humidified chamber. Subsequently, the stamp was washed with PBS and dried with nitrogen gas. At the same time, glass bottom Petri dishes (FD-35, http://www.wpiinc.com) were silanized using a 2% solution of 3-glycidoxypropyl trimethoxysilane in ethanol^47^. The protein-coated PDMS was placed in the middle of the 3-glycidoxypropyl trimethoxysilane-coated glass dish and incubated for 30 min at room temperature. Afterwards, the position of the PDMS stamp was labelled on the back of the glass dish and the stamp was detached from the glass. For backfilling the remaining area, a PDMS chamber with a square central opening was placed on top of the μCP-modified field, AlexaFluor568-labelled fibronectin (50 μg ml^−1^) was pipetted into the chamber and incubated for 1 h in the dark at room temperature. Finally, the PDMS chamber was removed from the glass, washed with 10 ml PBS and stored in PBS at 4 °C. After transfection with Xcad-11-EGFP using FuGene HD, cells were incubated for 16 h at 37 °C. Afterwards, cells were trypsinized and reseeded onto microstripe patterns, incubated for 4 h and finally fixed with 4% PFA for 30 min at room temperature. AFM imaging demonstrated that using a concentration of 20 μg μl^−1^ for laminin for stamp coating and 50 μg ml^−1^ for fibronectin backfilling yielded microstripe patterns with minimal topographic variation.

### Live-cell TIRF imaging

HeLa cells were grown in 25 cm^2^ culture flasks in RPMI medium until reaching 80% confluence. Afterwards, they were transfected with 10 μg μl^−1^ Xcad-11-EGFP and 2 μg μl^−1^ Vinculin-mCherry plasmid using FuGene HD. After overnight incubation at 37 °C, cells were reseeded on fibronectin-coated glass bottom Petri dish in CO_2_-independent Medium (Invitrogen). After 1 h TIRF images were acquired with an iMIC microscope (Till Photonics) and an APON 60XOTIRF objective (Olympus). All images were processed and analysed with ImageJ (http://rsbweb.nih.gov/ij/).

## Additional information

**How to cite this article:** Langhe, R. P *et al.* Cadherin-11 localizes to focal adhesions and promotes cell–substrate adhesion. *Nat. Commun.* 7:10909 doi: 10.1038/ncomms10909 (2016).

## Supplementary Material

Supplementary Information Supplementary Figures 1-14

Supplementary Movie 1 Time lapse movie of HeLa cells. HeLa cells were co-transfected with Xcad-11-EGFP and vinculin-mCherry and incubated overnight and reseeded on fibronectin. After 1 h TIRF microscopy images were recorded every 5 sec. Xcad-11 and vinculin colocalize at focal adhesions.
