# Supplementary material for: Cadherin-11 localizes to focal adhesions and promotes cell–substrate adhesion
Source: Nat Commun. 2016 Mar 8;7:10909. doi: 10.1038/ncomms10909 (PMC4786774; doi:10.1038/ncomms10909)
Supplement: Supplementary Information — Supplementary Figures 1-14 [file ncomms10909-s1.pdf]

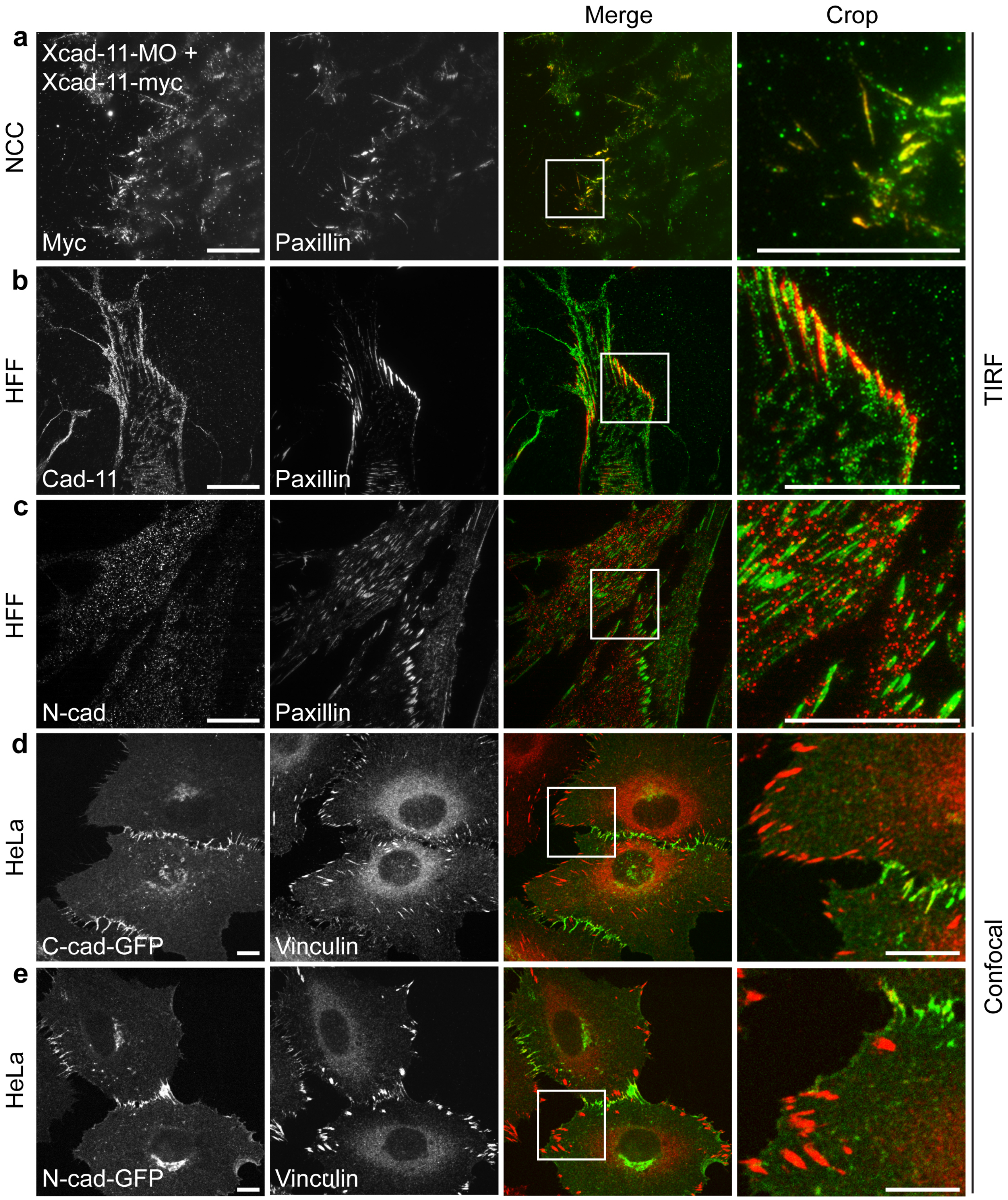

Supplementary Figure 1: ***Subcellular characterization of Xcad-11, N-cadherin and C-cadherin.***

(a) NCC explants co-injected with Xcad-11-MO and Xcad-11-myc and immunostained for myc (Xcad-11) and paxillin. TIRF images demonstrating co-localization of Xcad-11 with paxillin in FA at the cell-substrate interface. Co-immunostaining of endogenous (b) cadherin-11 or (c) N-cadherin with paxillin in HFF cells. TIRF images revealed co-localization of cadherin-11 but not N-cadherin with paxillin. HeLa cells were transfected with (d) C-cad-GFP or (e) N-cad-GFP, immunostained for vinculin and imaged by confocal microscopy. Neither C-cad-GFP nor N-cad-GFP co-localizes with vinculin. Scale bars: 10 μm.

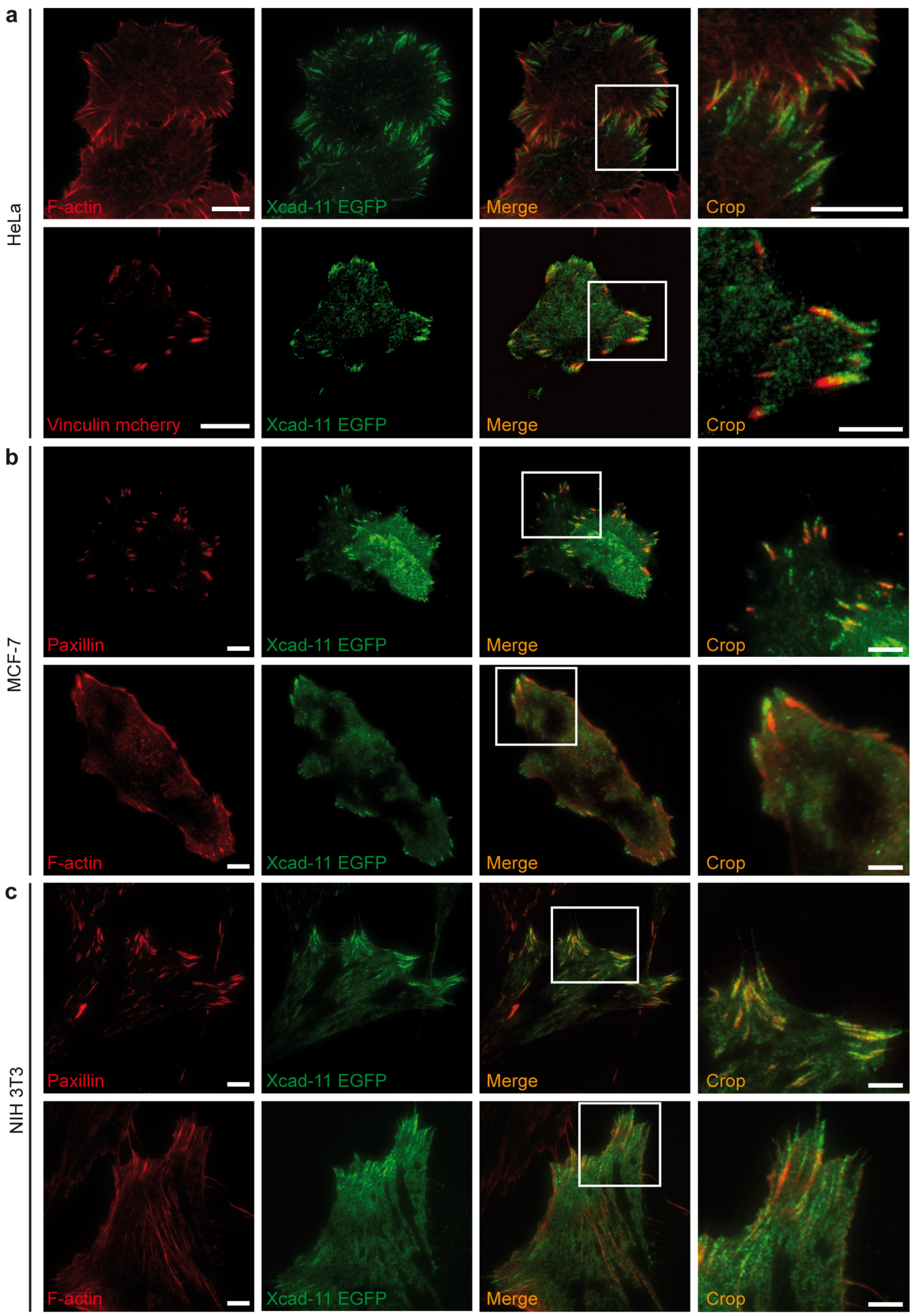

Supplementary Figure 2: ***Subcellular characterization of Xcad-11 at the cell-substrate interface of HeLa, MCF-7 and NIH 3T3 cells.***

(a) HeLa cells were transfected with Xcad-11-EGFP alone (upper row) or co-transfected with Xcad-11-EGFP and vinculin mCherry (bottom row). After incubation on fibronectin for 24-48 h, cells were fixed and stained for F-actin (upper row). TIRF images revealed partial co-localization of Xcad-11 EGFP with F-actin (upper row) and vinculin (lower row) at FA. (b) MCF-7 cells and (c) NIH 3T3 cells were transfected with Xcad-11-EGFP, immunostained for paxillin or phalloidin-stained to visualize F-actin and analyzed by TIRF microscopy. In both cell lines Xcad-11-EGFP partially co-localizes with paxillin and F-actin in FAs at cell edges. Scale bars: 10 μm.

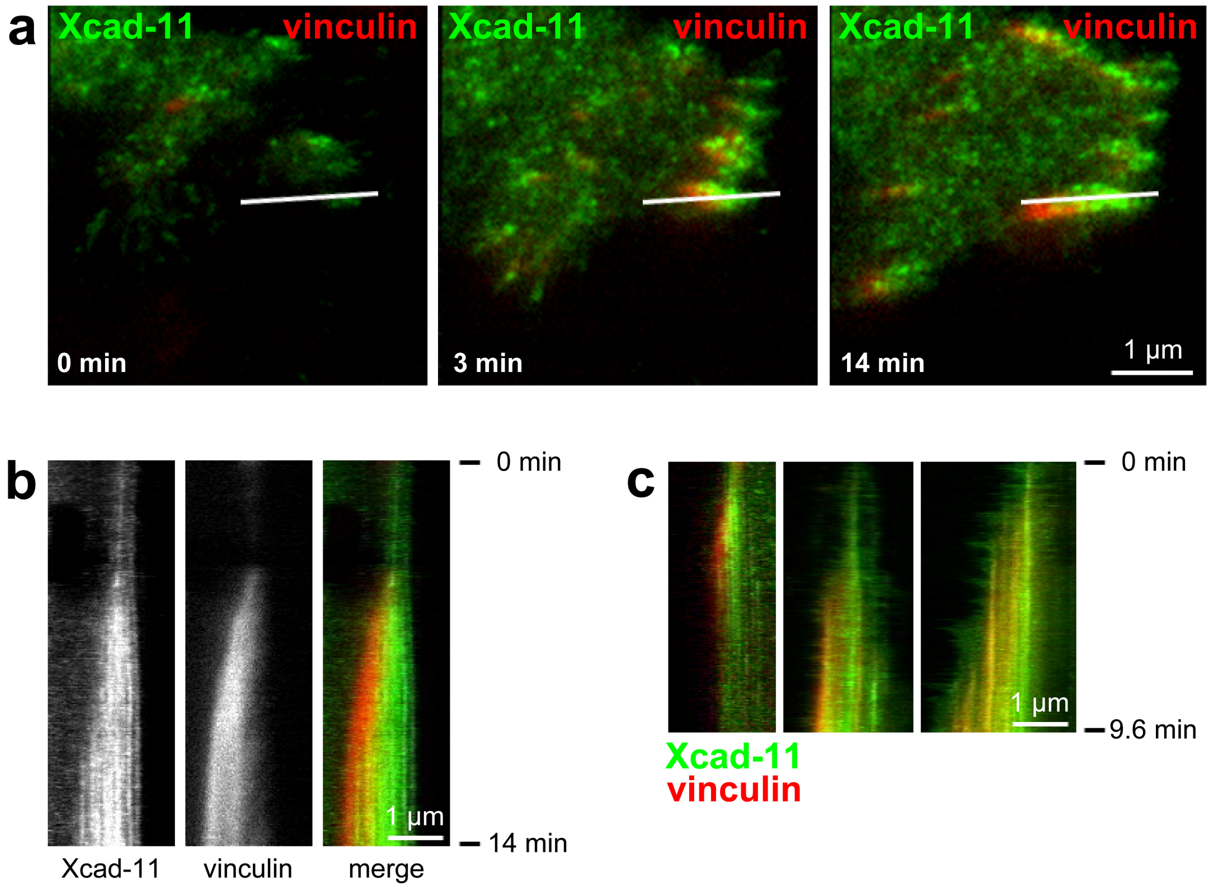

Supplementary Figure 3: ***Kymograph analysis of newly forming FA.***

HeLa cells were co-transfected with Xcad-11-EGFP and vinculin-mCherry, incubated overnight and reseeded on fibronectin. (a) Crops from a TIRF microscopy time lapse series recorded starting 1 h after reseeding. The white line indicates the position of the kymographs shown in (b). (c) Additional kymographs generated along emerging FA contacts at the cell periphery.

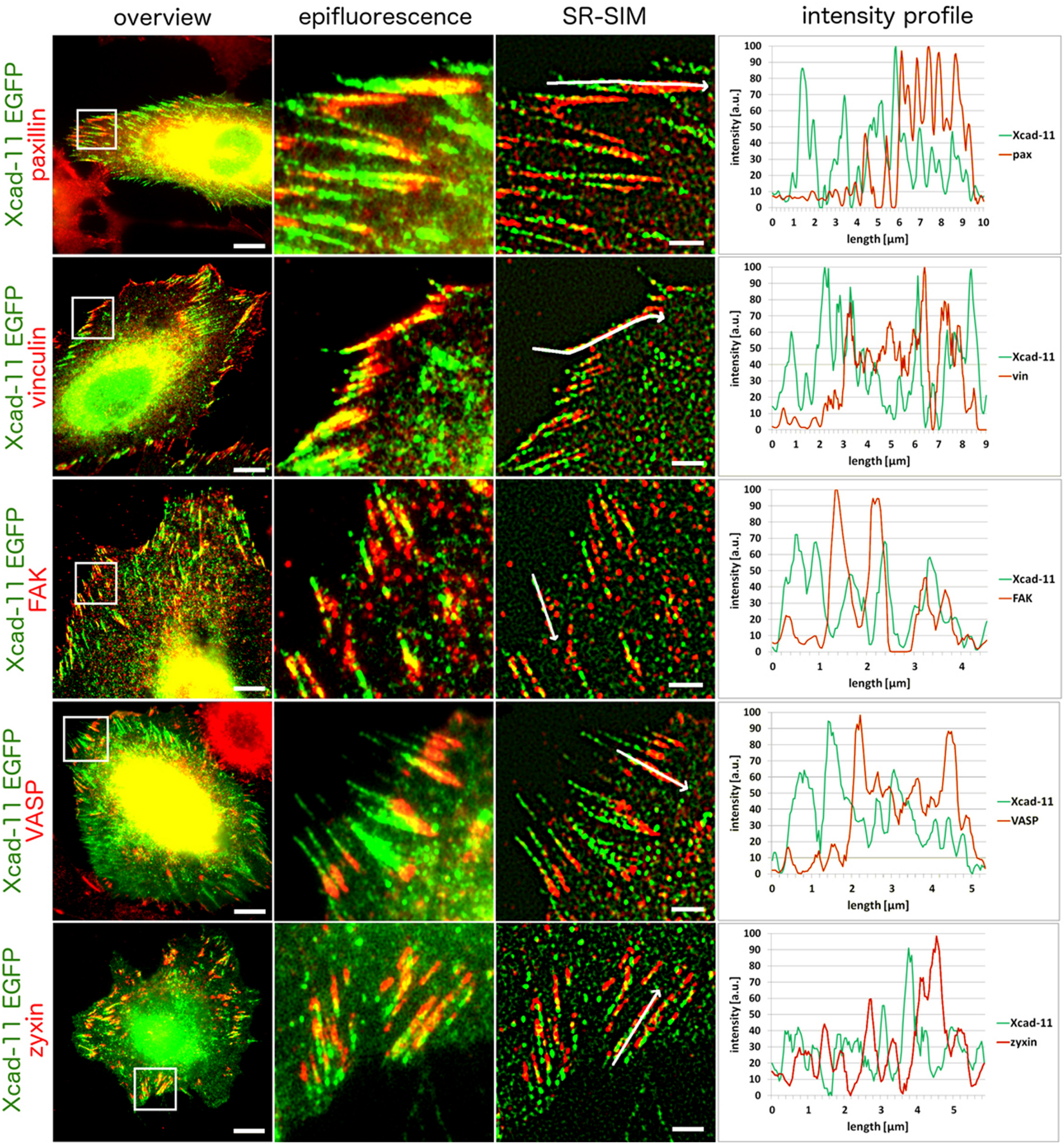

Supplementary Figure 4: ***Superresolution Structured Illumination Microscopy (SR-SIM).***

HeLa cells were transfected with Xcad-11-EGFP and immunostained for paxillin, vinculin, FAK, VASP, and zyxin. Intensity profile analysis revealed that Xcad-11 typically locates more distally in FA towards the cell margin, whereas paxillin, vinculin, FAK, VASP, and zyxin accumulate more proximally. Scale bars: 10 μm (overview), 2 μm (SR-SIM).

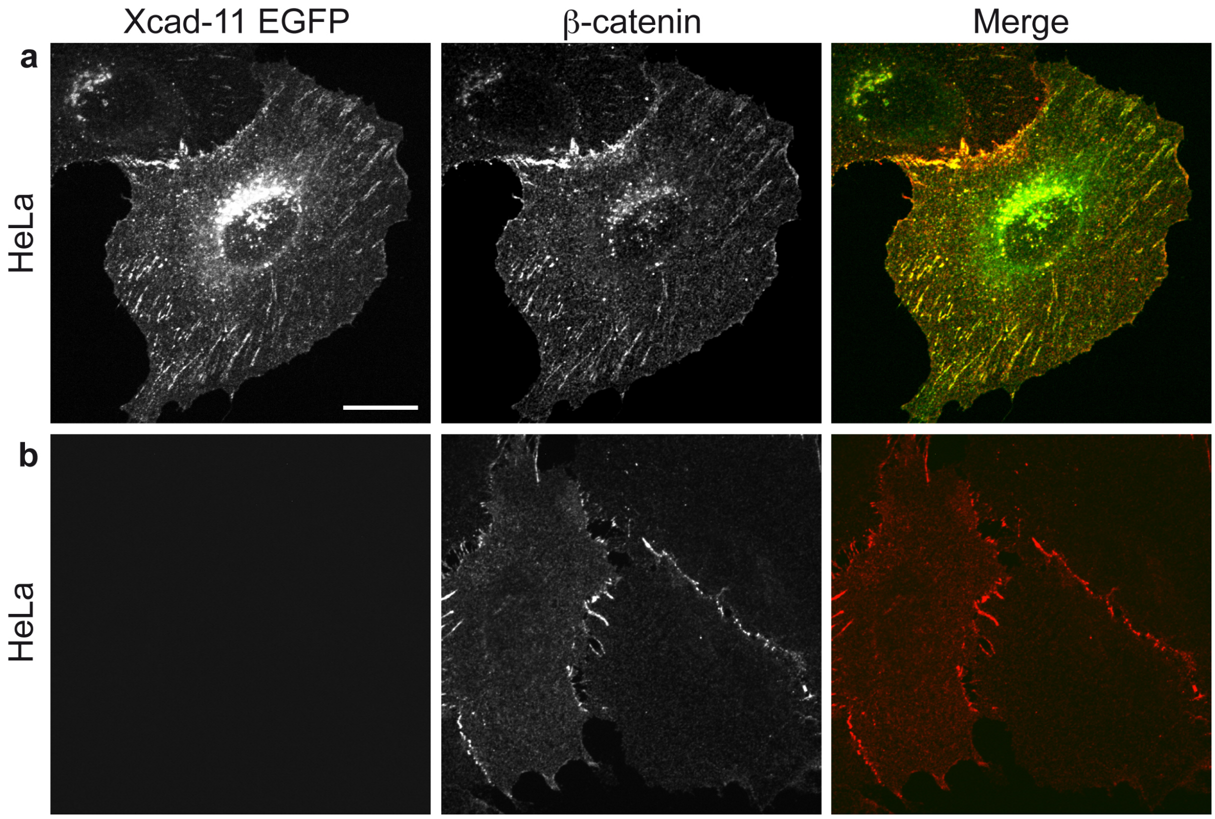

Supplementary Figure 5: ***Xcad-11 co-localizes with β-catenin in focal adhesions.***

(a) HeLa cells were transfected with Xcad-11-EGFP and immunostained for β-catenin. Confocal microscopy images revealed co-localization of Xcad-11 and β-catenin at cell-cell contact sites and in FA. (b) Untransfected HeLa cells displayed a prominent β-catenin localization at cell-cell contact sites. Scale bar: 20 µm.

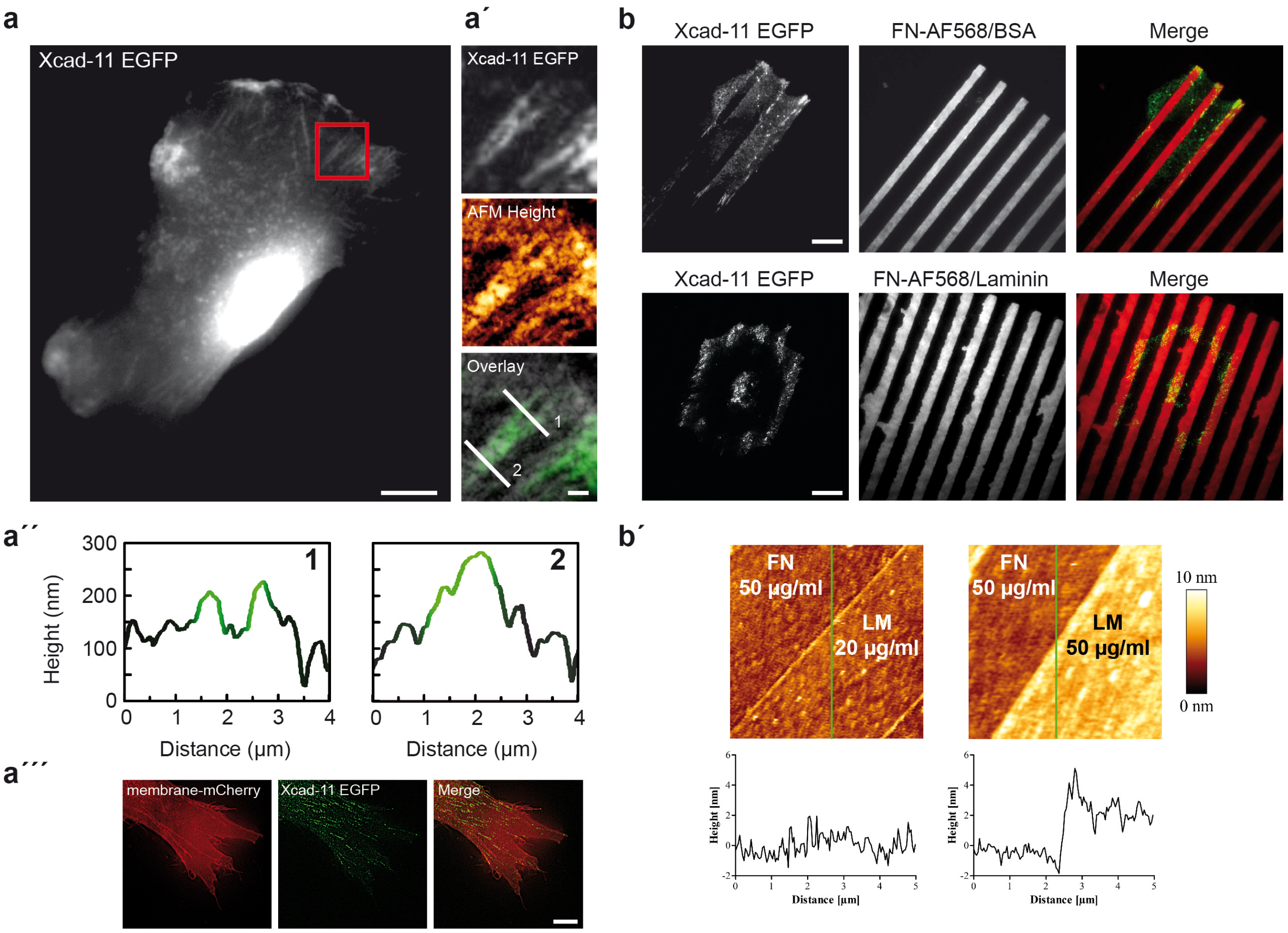

Supplementary Figure 6: ***Xcad-11 localization to FA is specific for fibronectin substrates.***

(a) Fluorescence image of an inverted HeLa cell expressing Xcad-11-EGFP. The area indicated by the red rectangle was scanned with AFM. (a´) Corresponding fluorescence, AFM height and overlay images. (a´´) Height profiles along cross sections 1 and 2 as indicated in (a´) are overlaid with the corresponding fluorescence intensities for better affiliation of height profile peaks and fluorescence intensities. (a´´´) TIRF microscopy image of a non-inverted Hela cell expressing membrane-mCherry to demonstrate homogeneous membrane distribution at the cell-substrate interface. (b) Fluorescence images of HeLa cells transfected with Xcad-11-EGFP and seeded on alternating fibronectin-AlexaFluor568/BSA and fibronectin-AlexaFluor568/laminin microstripes. HeLa cells form Xcad-11-EGFP positive FA selectively on fibronectin but not on laminin or BSA microstripes. (b´) AFM height images (5 x 5 µm^2) of fibronectin/laminin strip patterns at two different laminin concentrations (20 and 50 µg/µl). Green lines indicate the position of height profiles shown below the AFM images. Using a laminin concentration of 20 µg/µl for PDMS stamp coating and a fibronectin backfill concentration of 50 µg/µl generates surfaces with minimal topographic variation. Scale bars: (a) 10 µm, (a´) 1 µm, (a´´´) 2 µm, (b) 10 µm.

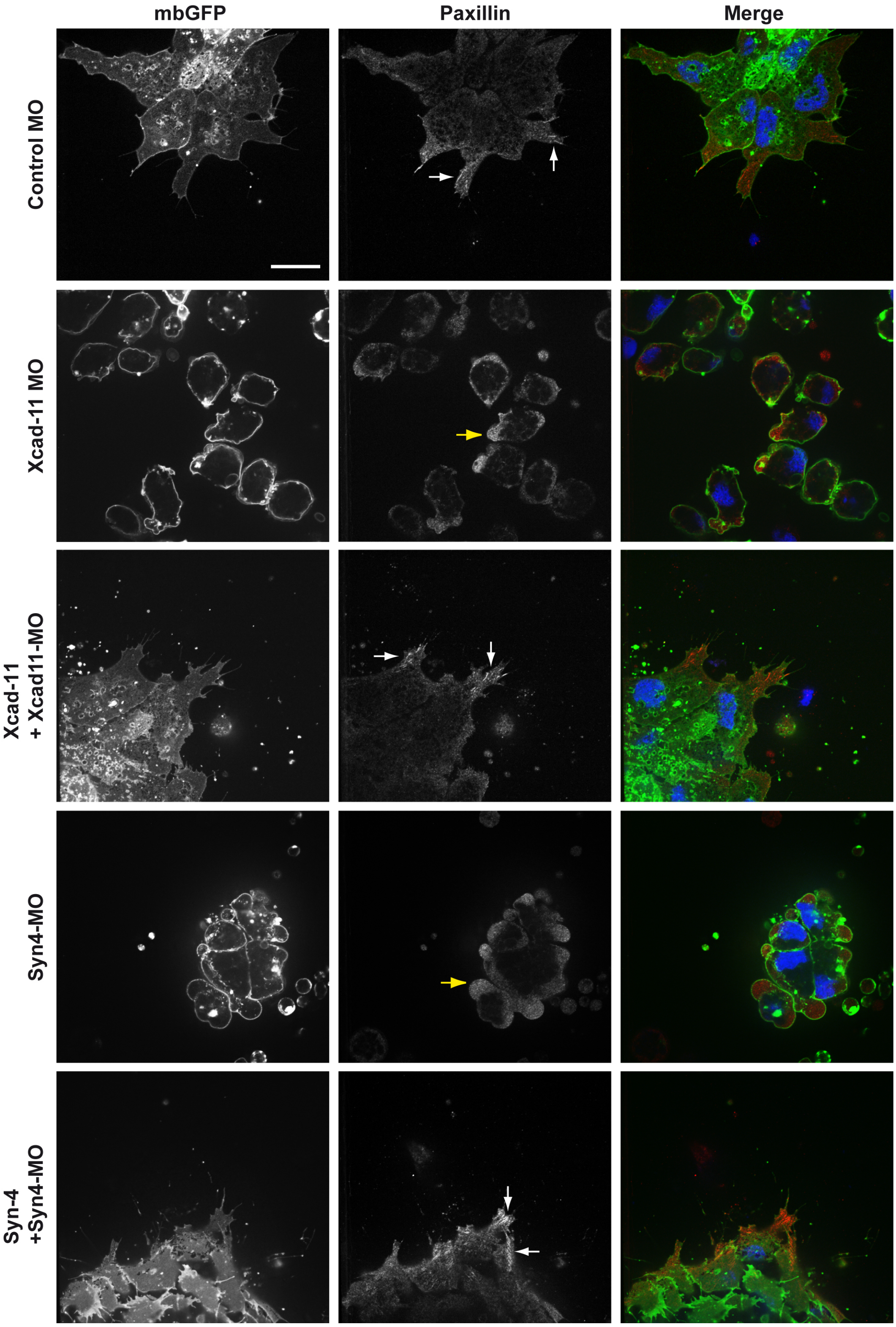

Supplementary Figure 7: ***Xcad-11 and Syn-4 promote focal adhesion formation.***

Immunostaining of paxillin on NCC explants injected with control MO, Xcad-11 MO, Xcad-11 together with Xcad-11 MO, Syn-4 MO or Syn-4 together with Syn-4 MO. mbGFP was used to label plasma membranes. Control MO injected NCC spread on fibronectin, form cell protrusions and show paxillin staining in FAs at the leading edge. In contrast, Xcad-11 and Syn-4 morphant NCC display only diffuse paxillin staining in the cytoplasm as well as in blebs and lose the ability to form cell protrusions. Co-injection of Xcad-11 or Syn-4 rescues FA and cell protrusion formation. Scale bar: 20 µm.

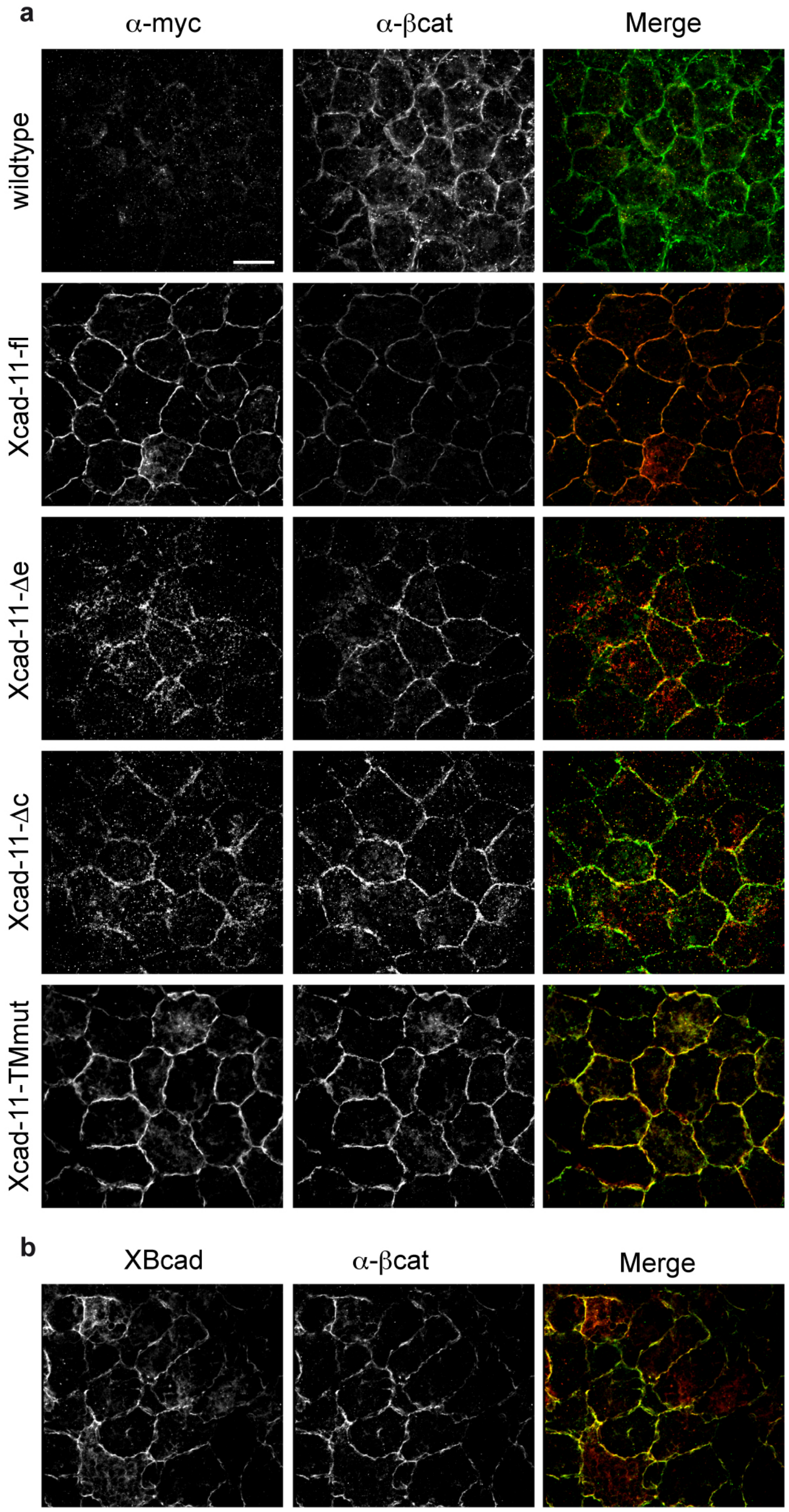

Supplementary Figure 8: ***Subcellular localization of the different Xcad-11 deletion constructs analyzed in Xenopus animal caps*****.**

(a) Embryos were injected with full length (fl) Xcad-11, Xcad-11 Δc, Xcad-11 Δe and Xcad-11 TM mut. Immunostaining for the myc-tag of the different Xcad-11 constructs on dissected animal caps revealed prominent localization at cell-cell contacts of all constructs as demonstrated by co-immunostaining with β-catenin (β-cat). (b) Co-immunostaining of XB-cadherin (XBcad) and β-catenin served as positive control. Scale bar: 10 μm.

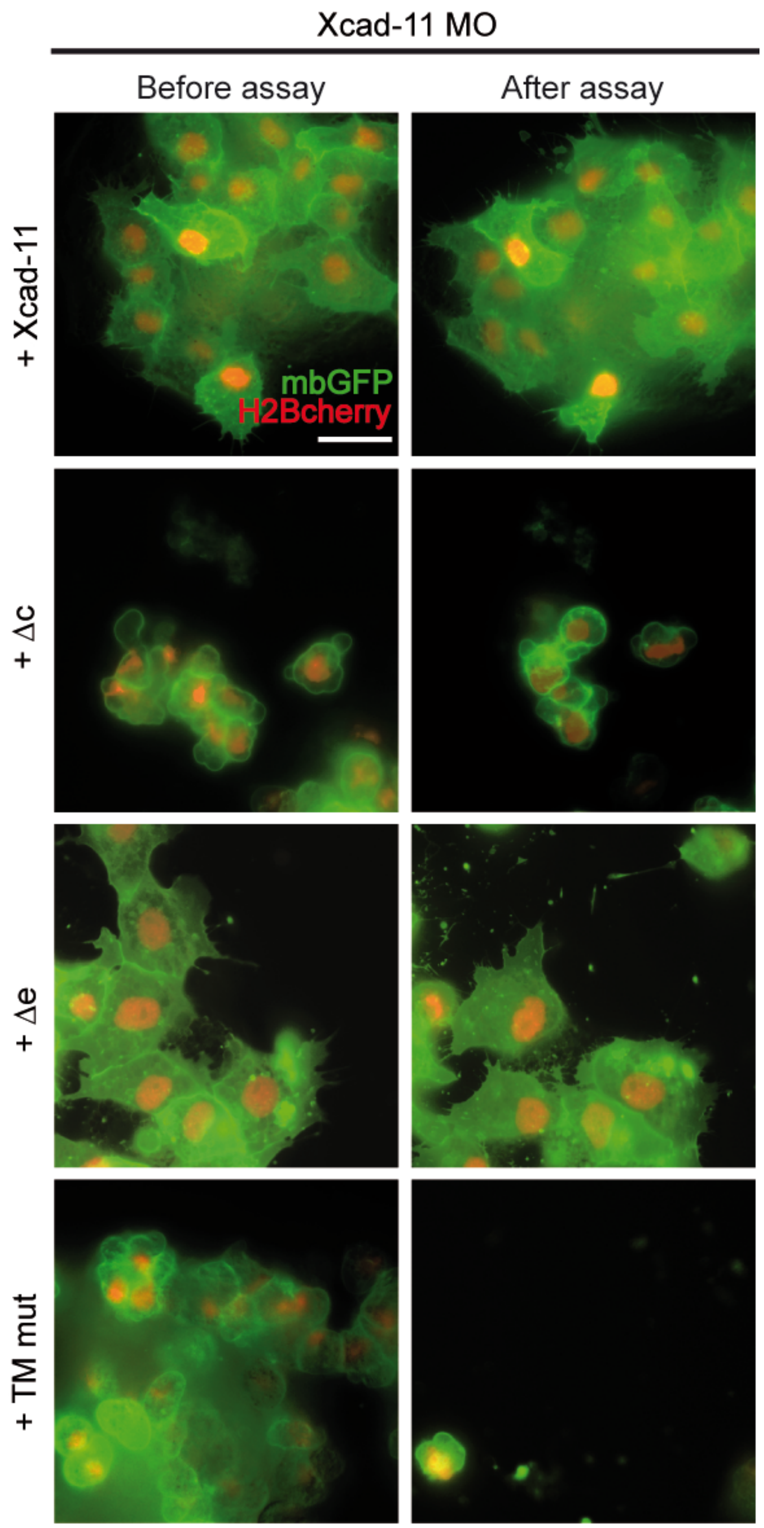

Supplementary Figure 9: ***Rescue experiment with different Xcad-11 deletion constructs in the flipping assay.*** Embryos were injected with Xcad-11 (75 pg), Xcad-11 Δc (75 pg), Xcad-11 Δe (75 pg) and Xcad-11 TM mut (75 pg) along with Xcad-11 MO (16 ng). mbGFP (100 pg) and H2Bcherry (100 pg) were used to label plasma membranes and nuclei, respectively. Fluorescence images collected at the same substrate position before (right column) and after (left column) the flipping assay. NCC cells injected together with Xcad-11 MO and full length Xcad-11 or Xcad-11 Δe exhibit cell spreading along with strong cell adhesion to fibronectin. In contrast, co-injection of Xcad-11 MO with Xcad-11 Δc or Xcad-11 TM mut leads to reduced cell adhesion to fibronectin. Scale bar: 20 µm.

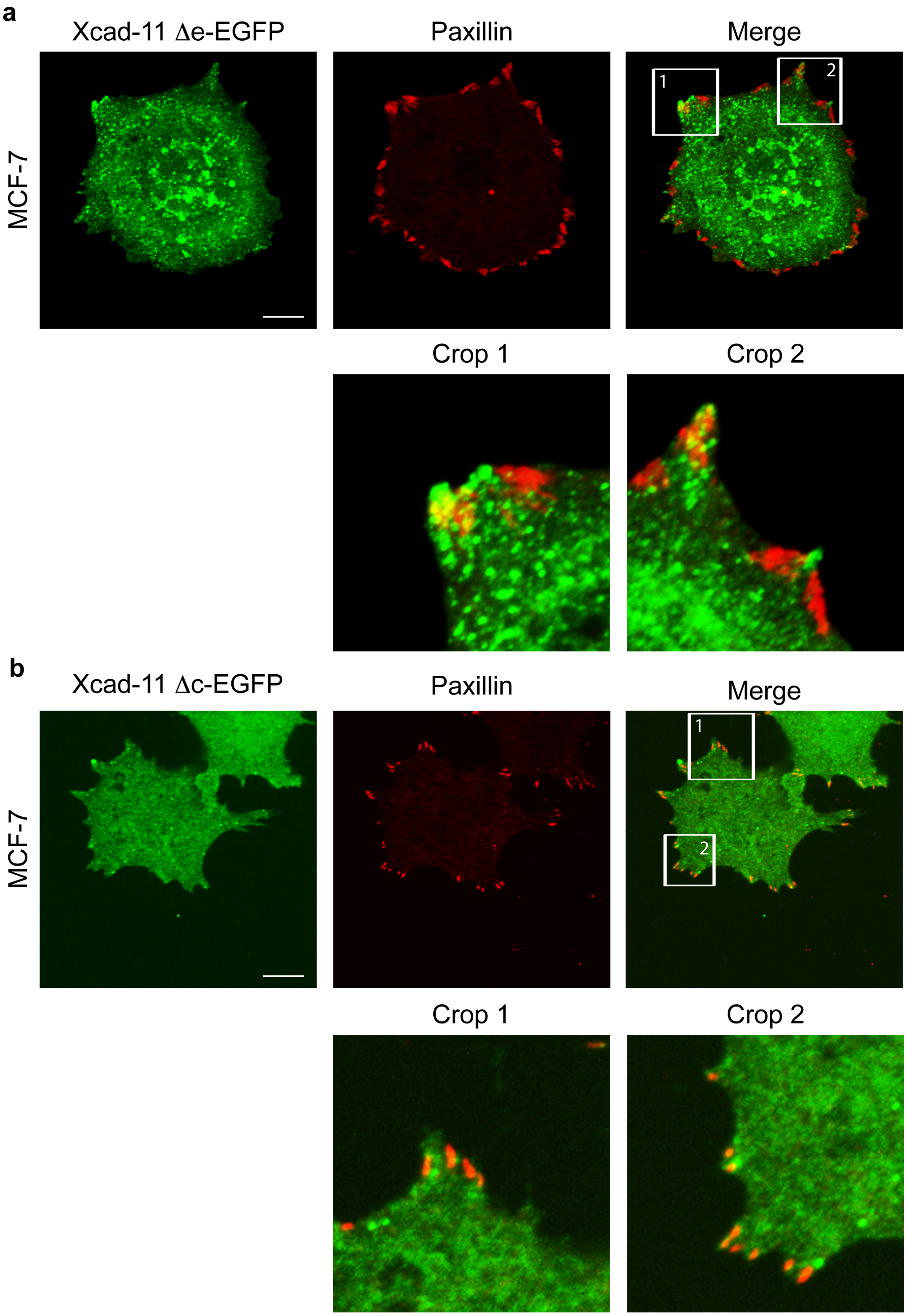

Supplementary Fig. 10: ***Subcellular localization of Xcad-11 Δe-EGFP and Δc-EGFP in MCF-7 cells.***
MCF-7 cells were transfected with (a) Xcad-11 Δe-EGFP or (b) Xcad-11 Δc-EGFP, immunostained for paxillin and analyzed by confocal microscopy. Xcad-11 Δe-EGFP partially co-localizes with paxillin whereas Xcad-11 Δc-EGFP displays a homogeneous localization. Scale bars: 10 μm.

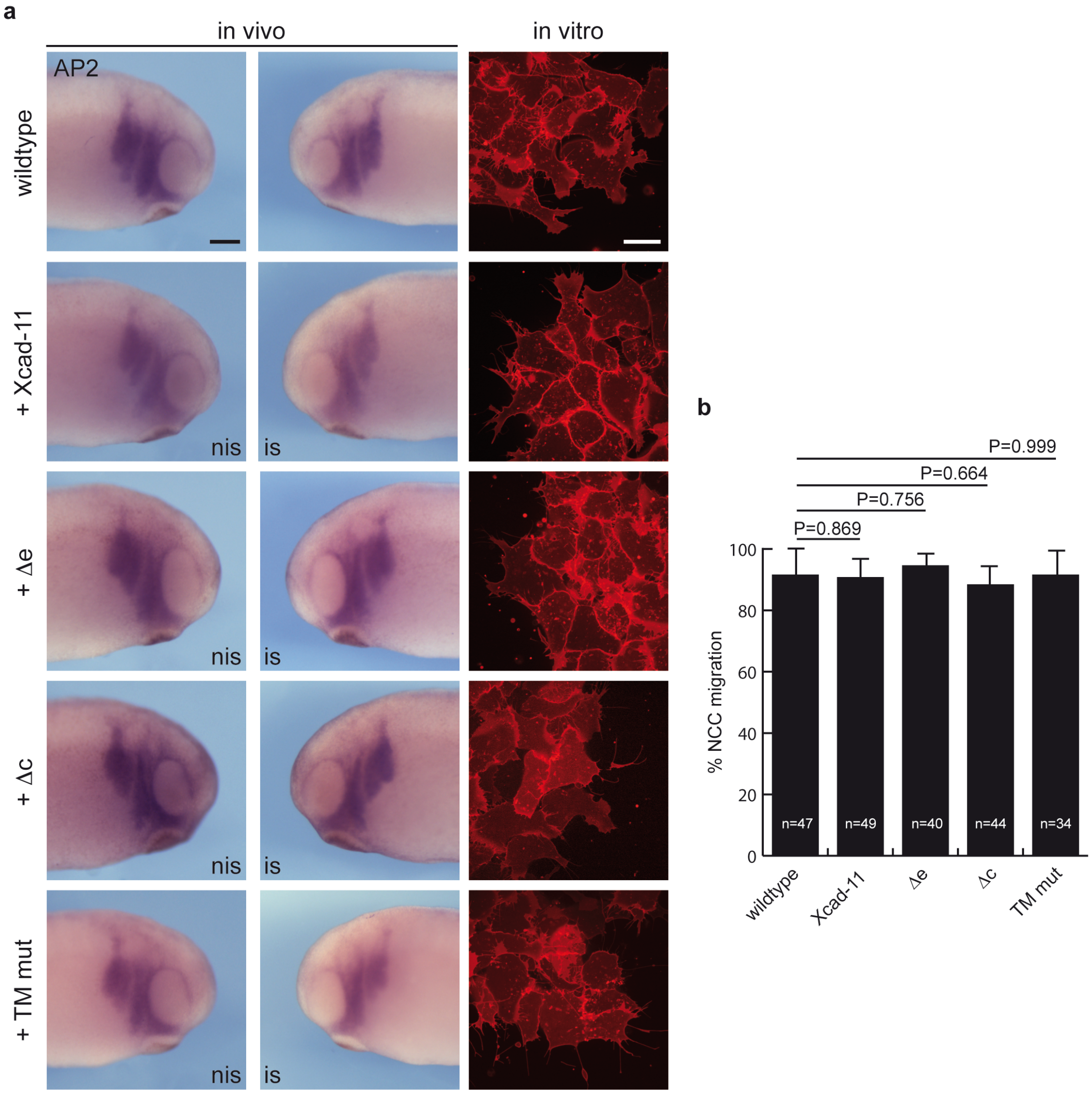

Supplementary Figure 11: ***Overexpression of the different Xcad-11 constructs does not block NCC migration in vivo or cell protrusion formation in vitro.***

(a) Lateral view of *Xenopus* NCC at stage 26, analysed by whole-mount ISH for the specific NCC marker AP-2. Embryos were injected at 8-cell stage with Xcad-11 (100 pg), Xcad-11 Δc (100 pg), Xcad-11 Δe (50 pg) and Xcad-11 TM mut (100 pg), respectively. Nis: non-injected side (left column); is: injected side (middle column). NCC migration is not affected by co-injection of each Xcad-11 construct. Scale bar: 200 µm. NCC explants co-injected with mbCherry and the indicated Xcad-11 constructs (right column). Cell protrusion formation is not affected by co-injection of each Xcad-11 construct. Scale bar: 20 µm. (b) Statistics for whole-mount ISH (mean ± S.D.). n = number of embryos. Results of at least three independent experiments were averaged and statistical significance was analysed by student's T-Test.

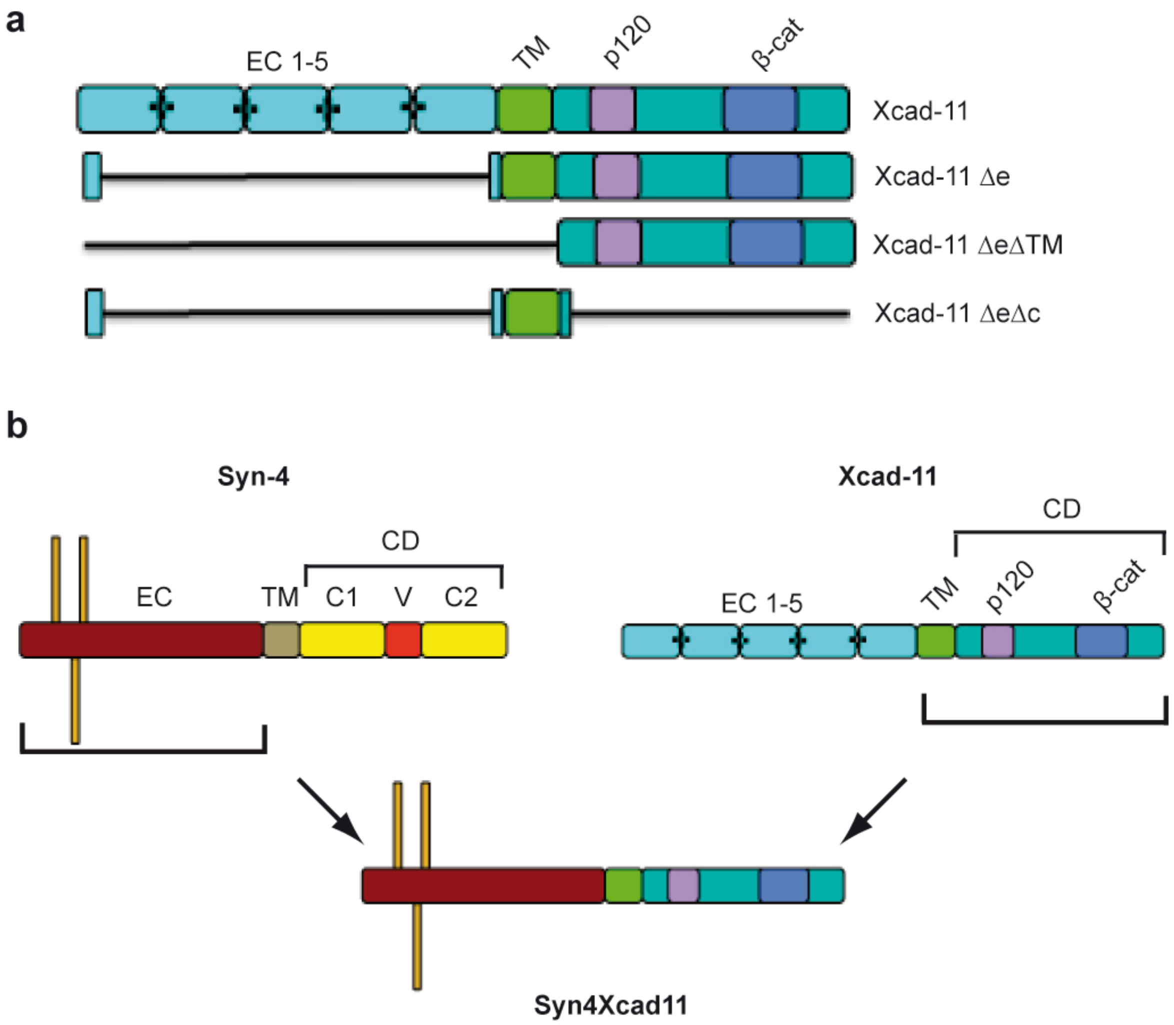

Supplementary Figure 12: ***Schematic representation of different Xcad-11 constructs.***

(a) Schematic representation of different Xcad-11 constructs used for Co-IP experiments with Syn-4. EC: extracellular domain, TM: transmembrane domain, p120: p120-binding site, β-cat: β-catenin binding site. (b) Schematic representation of the chimeric Syn4Xcad11 construct. In the Syn-4 protein, C1, V and C2 designate cytoplasmic sub-domains. V is a variable domain. The Syn-4 extracellular (EC) domain was amplified by PCR and ligated with the TM and cytoplasmic domain of Xcad-11.

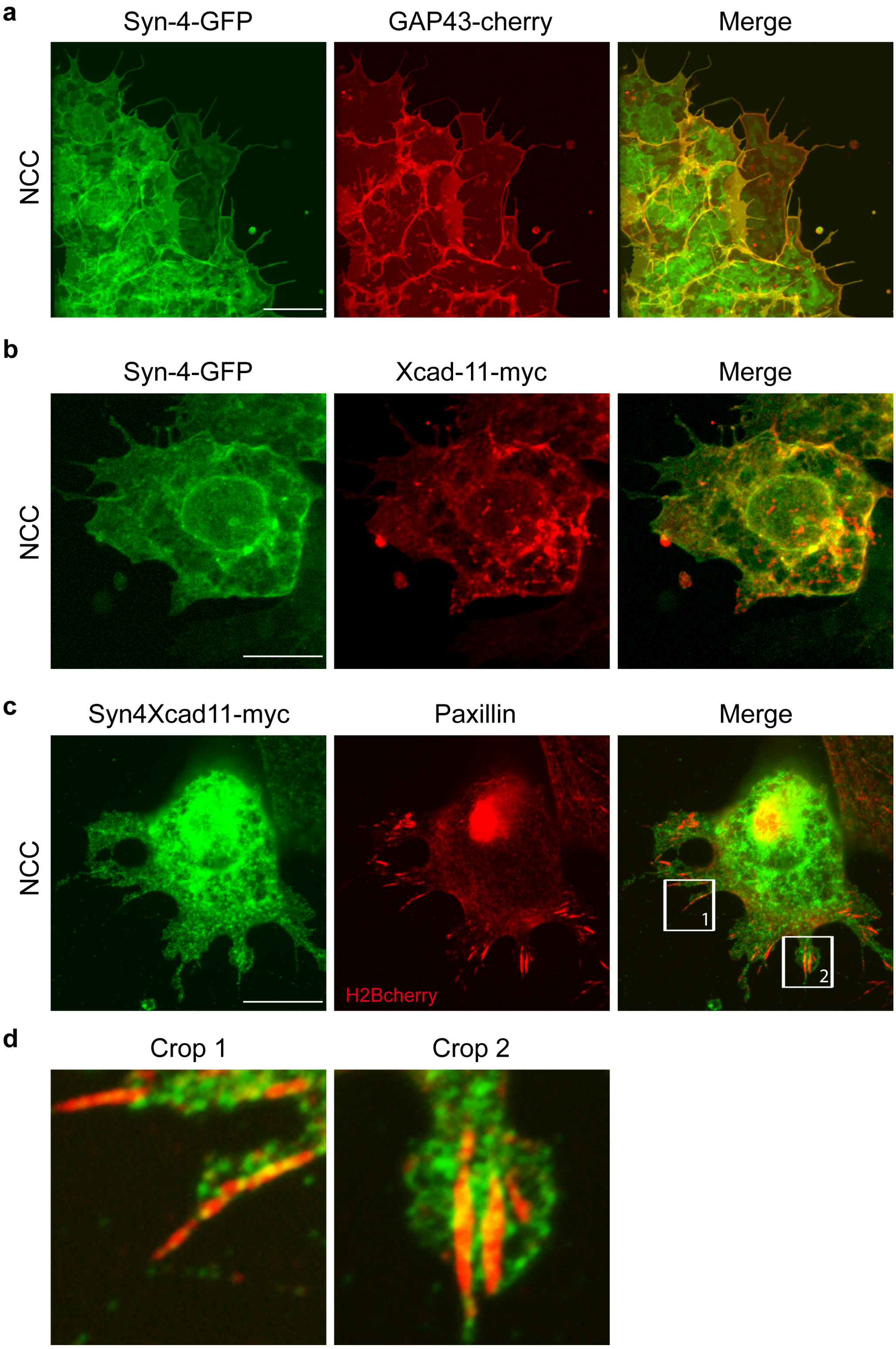

Supplementary Fig. 13: ***Subcellular localization of Syn-4-GFP and Syn4Xcad-11 in NCC.***

(a) NCC explants co-injected with Syn-4-GFP and GAP43-cherry and analysed by confocal microscopy. Syn-4-GFP is homogeneously localized within the entire NCC. (b) NCC explants co-injected with Syn-4-GFP and Xcad-11-myc and immunostained for myc (Xcad-11). (c) NCC explants co-injected with Syn4Xcad-11-myc as well as H2Bcherry and immunostained for myc (Syn4Xcad-11) and paxillin. Confocal images demonstrating partial co-localization of Syn4Xcad-11with paxillin in FA at the cell-substrate interface. (d) Cropped areas as indicated in (c). Scale bars: 20 μm.

Figure 3a

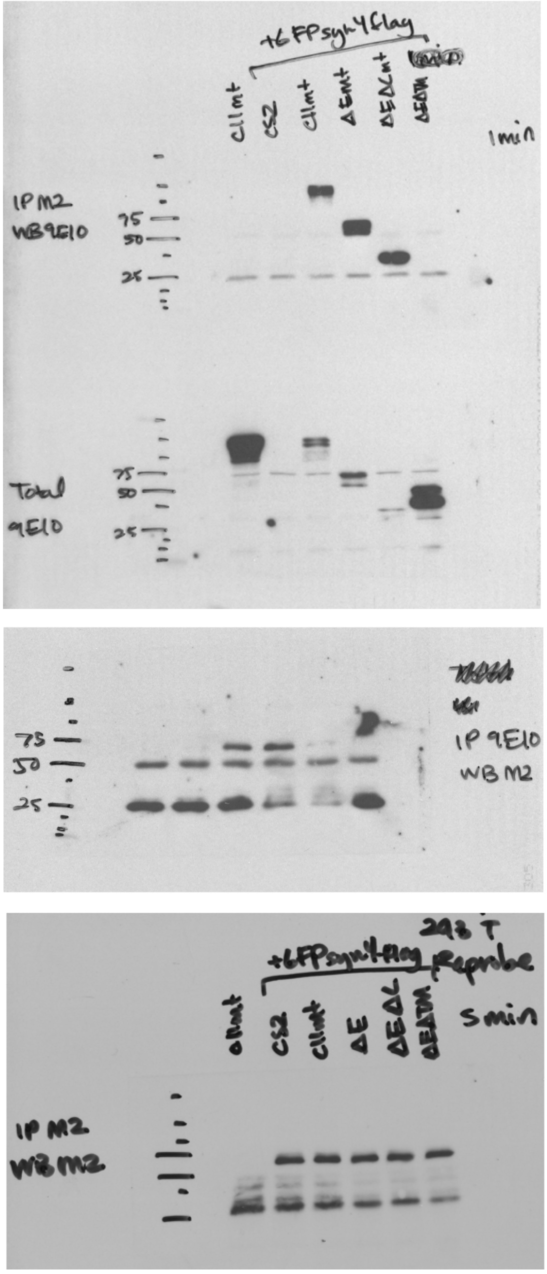

Figure 3b

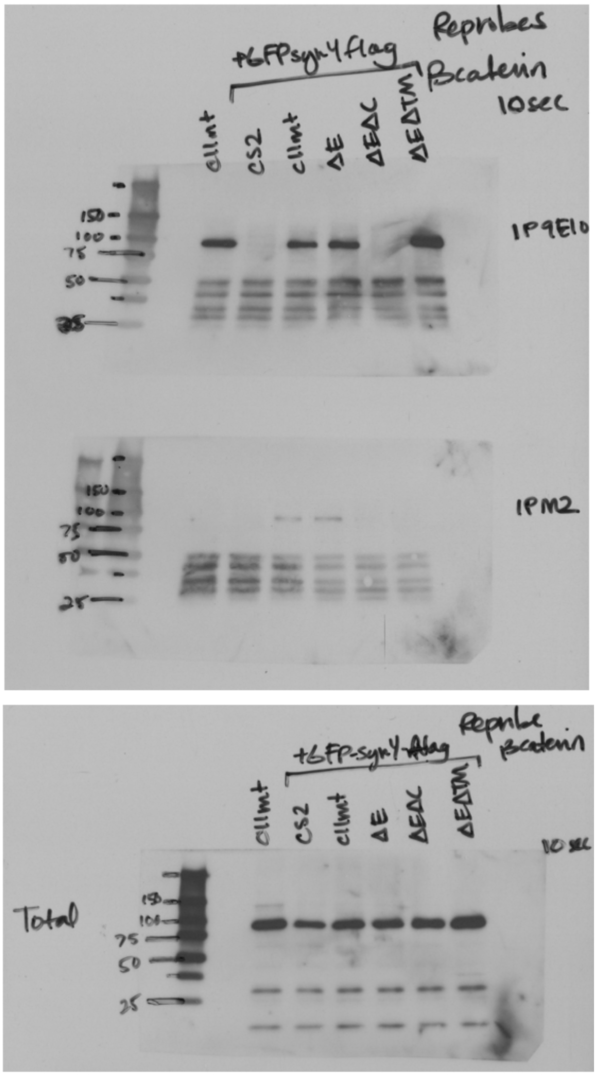

Supplementary Figure 14: ***Full scans of Western blots.***
